# Internet Medical Usage in Japan: Current Situation and Issues

**DOI:** 10.2196/jmir.3.1.e12

**Published:** 2001-03-17

**Authors:** Haruyuki Tatsumi, Hiroaki Mitani, Yasuo Haruki, Youichi Ogushi

**Affiliations:** ^1^Department of AnatomySapporo Medical UniversityJapan; ^2^Japan Internet Medical AssociationJapan; ^3^Department of Medical InformaticsTokai University School of MedicineJapan

**Keywords:** Internet, Quality Information, Ethics Codes, Japan, Certification System

## Abstract

Internet use by physicians and patients has become very popular in Japan. Fifty percent of physicians use the Internet to search for medical and other information. Over the past year, 22% of patients used the Internet to obtain medical information. Because there are no restrictions within Japan on using Web sites to advertise medical treatment, information can be freely sent out, and over the past two or three years this practice has increased dramatically. Internet medical information provides information about illnesses and medications, and it helps improve the quality of life of patients and families.

Yet, depending on the content of the information provided and the way this information is used, there is a potential negative side as well. On principle, users are responsible for the way information is used, but there is a need for information providers to consider users' safety and to make the information effective for use. Because there is no absolute standard for evaluating the value of medical information, it is necessary to establish a system that opens a dialogue with society and that continuously accumulates high-quality information through the collection of various evaluations, rather than rely on an established authority. For industries and organizations related to commercial pursuits, in particular, it is most effective to establish their own codes for ethical conduct, rather than rely on governmental regulations. At the same time, it is important to have a confirmation function to evaluate how goals set by the outside are being implemented.

Aiming at establishing a framework for the Internet medical usage, the Japan Internet Medical Association (JIMA) was founded in 1998 by medical professionals, lawyers, researchers, consumer representatives, patients and their families. We propose a system that would combine feedback from users, who would take on the role of evaluators of the implementation of an ethical code, with a displayed mark that verifies the identity of the Web site. Objective evaluation of information is needed to ensure that users have the power to make choices. Medical experts or patient and family groups would assist in this task. The development of medical care will be promoted through patients and physicians' working together in the accumulation of shared resources for good medical care information.

## Introduction

As the population with Internet access increases in Japan, there is a notable trend toward the use of this new information communication medium in the fields of medicine and welfare. The words "e-commerce" and "e-business" may be taken as indications of a new human economic activity based on the latest information technology medium. "E-health" is now a commonly used word in Europe and America, but in Japan it still does not have a very familiar ring. One of the special features of the medical care environment in Japan is the health insurance system, which ensures that all citizens can receive basically equal medical treatment. Under this system, the medical care needs of a broad population are served by institutions that provide medical treatment, from regional medical clinics that provide primary care to highly specialized hospitals that provide comprehensive care. In the near future, however, there will be changes in the way people think about medical care for the nation's citizens.

Examples include litigation actions brought on by the treatment of AIDS and problems of recently increasing medical malpractice. In response to such incidents, patients and the population in general are looking at medical care with a critical eye. Another example is the increased use of the term "informed consent," which represents the emphasis placed on patients' rights and trends in the release of information, as seen in the trend toward disclosing of patient charts. These changes have just begun, but in the future, as patients become aware of their rights, it is expected that these trends will become stronger. Another societal factor is the rapid aging of the population. The increase of the older population will increase the nation's financial burden of medical expenses. As a result, the foundations of the insurance system, which has provided low-cost medical care to citizens, will be increasingly threatened.

## The Current Situation and Issues

### Background

With these changes in the social environment taking place, medical policy is needed that both contains the nation's medical expenses and provides high-quality, high-efficiency healthcare. Efforts should be made to prevent illnesses so that people can live long, healthy lives, and efforts should be made to increase the quality of life through the appropriate control of illness. In the past, under the national insurance system, one could obtain medical treatment, with no great disparity in the quality of treatment, regardless of location. Patients would generally accept the provided treatment passively, without asserting themselves. Now, however, this passive attitude is changing as issues of protecting independence in medical care are being raised. Patients increasingly want to make their own decisions about methods of therapy and care.

One significant factor driving this change is the increased convenience of accessing medical treatment information through the Internet. In other words, as the Internet increases the demand for information about medical treatment and health that is needed by patients and families [1], the medical institutions and other providers supplying such information will provide an environment in which it is easier to send information, thereby accelerating the flow of released information [2].

### Access to the Internet in Japan

According to a study supported by a research grant of Japan's Ministry of Health and Welfare, as of January 2000, 34% of 1021 medical institutions were providing some form of information about their work or the medical treatment they provide through a Web site [3] (see appendix for the full study). In addition, 50% of physicians were using the Internet. Of the physicians using the Internet, 65% were "searching for medical/treatment information," and 56% were "corresponding." Furthermore, with regard to making information about the physician's medical institution accessible, physicians were in favor of making basic information about hospitals and clinics available, 87% agreed to having physicians' names made available, and 80% or more thought information about physicians themselves, such as field of specialization, medical society certification, and university, should be made public.

As for what benefits for patients could be expected from the release of information about medical care institutions, 78% thought that it would be "useful to patients for the selection of medical care institutions," and 70% thought that it would make "going to medical care institutions easier." With regard to how the accessibility of information about their own illness would change the relationship between physicians and patients, 62% thought it would "bring into being the right of patients to make their own decisions." Finally, 59% thought that "as the amount of information that can be shared increases, relationships of trust will further increase." From these numbers, we see that medical care institutions and physicians will look favorably on future trends toward the release of general information.

### Internet Use in Japan

What about the thinking of those who use medical information provided by medical care institutions and physicians? According to a study conducted in February 2000 supported by a grant of the Ministry of Health and Welfare (joint researcher, Sapporo Medical University professor, Haruyuki Tatsumi), the Japan Internet Medical Association (JIMA) found that, of the 1842 persons who were using the 29 institutions and were the object of the study, the proportion of patients/families using the medical institutions that were using the Internet was 645, or 35% [3]. At present, 13% of the Japanese population uses the Internet (Nielson NetRatings, December 1999). Compared to this figure, both patients and physicians have higher than average rates of Internet use.

As for use of Internet medical information by patients/families, 405 persons used medical information over the previous year. This figure was 63% of the Internet users and 22% of the total respondents. In their use of the Internet, 41% "obtained specialized information about an illness," 30% "obtained information about health control such as preventing illness," and 27% "obtained information about medications." With regard to future use of medical information, 67% said they would "like to obtain more specialized information concerning illness and health control," and 60% said they would "like to obtain more specialized information about effectiveness and side effects of medications." From these figures, we have concluded that patients and families have a strong desire to obtain specialized information about illnesses and medications.

Additionally, with regard to obtaining information about medical institutions, the items of interest relating to medical institutions that concerned the content of medical treatment and systems for providing such treatment were "information about examinations, diagnosis, and therapy." For these items, interest was high at 65%. Interest for an item relating to patient care, "explanation of an illness to the patient and family," was high at 75%. And, with regard to information about physicians, 71% wanted information about "particular fields of specialization" released.

From the perspective of a country such as the United States, where information about medical institutions and physicians was released early on, the meaning of such data may be hard to understand. According to the provisions of Japanese law (Medical Law No. 69), when medical institutions provide information about the content of treatment administered and their work to an unspecified, large number of people, it is considered advertising, and restrictions have been established for what information can be released. Medical law is currently being reexamined for the relaxation of advertising restrictions, but many restrictions will probably remain. The Internet, however, is exempt from these restrictions. Information provided through a Web site is not considered advertising and is allowed. This circumstance is one reason for the rapid establishment of Web sites by medical institutions over the last two or three years [4].

In this way, the increase in medical information provided over the Internet is a favorable development for patients and the population in general: it allows them to obtain information about their own health or illness, deepen their knowledge, and independently manage their own illness or health. By increasing the sources of information, and broadening the range of choices, it will be possible to find the most appropriate therapy or care [5]. In a questionnaire study conducted by JIMA in March 1999, of 502 persons among patients/families and citizens who use the Internet to obtain medical information, 83% said that medical information provided over the Internet was useful in improving daily life. There were various forms of use, such as homepage browsing, bulletin boards (electronic meeting rooms), mailing lists, and e-mail medical consultation. Of these respondents, 37% said that, as a result of using this information, they were "able to have a good understanding of medical treatment they were presently receiving," and 11% said that they were "prompted to go to the medical institution." Thus, the trend was one of positive evaluation.

### Quality of Health Information

With regard to opinions about the quality of the medical information provided, 68% said that the content of the information "was reliable," and a combined total of 32% said that it "was unreliable" or that they were "undecided." Reasons given for the unreliability of the information were that "there was no one to guarantee the information's reliability," "the content is for advertising," "sometimes it is not clear who is issuing it," "the information is slanted," and so on. When asked whether or not using medical information in the past had actually led to problems or trouble, 6% said that it had. Concrete examples included "advertising mail about related products were continually sent" and "the actual treatment by the physician was different from my expectations."

Although no serious example has been set up to now, there is concern about the use of medical information. Fifty-seven percent said that "the use of bad information or the misuse of information could endanger the user," 45% said that "the issuance of information being used for commercialism or for profit-making competition was a concern," 43% said that "it was hard to question the issuer's responsibility with regard to the content of information," and 24% said that "when the patients have too much information, many will actually have more trouble making decisions."

Perhaps because of these concerns, in response to an item on the current state of rapid developments in the use of Internet medicine, 42% believed that "some form of regulation is needed to ensure the safety of users," and 22% believed that "no regulation beyond current law is needed if users thoroughly follow the principle of accepting personal responsibility." From these results it can be seen that users of Internet medical information have a strong desire for specialized medical information pertaining to illnesses and health, but that the level of trust regarding the content of various information is not always high and that users have various concerns about the use of information.

With regard to the quality of Internet medical and health information, problems have appeared in Europe and America [6,7], and the same is true for Japan. Such information is issued either by individuals or organizations, for profit or not for profit, and from various standpoints and for various objectives. The control of quality is in various hands, and the level of control also varies. Information on medical treatment and health may affect the lives of people, and there is a need for high quality, yet there is no system in place for objectively evaluating it and providing users with helpful information. With the lack of such a system in place, a Web site was established in Japan that sent a hydrocyanic acid chemical compound over the Internet to a person who wanted to commit suicide, and in December 1998, an unfortunate incident occurred in which a young woman who used this substance died. Reacting to reports of such incidents, the general public has come to adopt a critical view.

To what extent can information from the Internet be trusted? With regard to this question, in the study mentioned above [3], JIMA selected 516 Web sites from the medical institute directory of the medical page of "YAHOO!JAPAN," a large search site, drawing from five categories: internal medicine, pediatrics, dermatology, behavioral health, and neurological surgery. Numerous specialist physicians from each discipline conducted evaluations of the medical information being provided. Judging the content of information provided, these physicians found that 7% of the sites "have problems." The reasons given included "there is a concern that ordinary people will use bad information," "information with inadequate verification is included," "it diverges from current standard medicine," "the content is slanted," and "there are errors in the article items." The category that was found to have the most problems was dermatology, and the one with the fewest problems was internal medicine. Whether 7% is considered to be many or few, the reality is that if a specialist in the same field finds problems in a medical institution's Web site, which ought to be reliable, it is due reason for exercising caution.

### Ethical Codes

As medicine progresses, the techniques and content of medical treatment change. And as information concerning medical treatment and health becomes more diversified, the volume of information provided via diverse media increases dramatically. Although these changes greatly benefit the user, the information becomes more diverse and voluminous; hence, it becomes difficult for the user to correctly understand the content and confirm authenticity [6,8]. To enable users to reap an even greater benefit from the accessibility and use of information from the Internet, a societal arrangement is needed to properly conduct the flow and use of information [7,9]. In the development of e-health, which first arrived in Europe and America, a code of ethics was created to define standards for conduct. This code of conduct is based on self-regulation by the companies and organizations that provide information and services, and it was developed so that users could safely use information. The e-Health Code of Ethics, Health on the Net Foundation (HON), Hi-Ethics, Health Insurance Portability and Accountability Act (HIPAA) were all created as the Internet was developing. In addition, the American Medical Association has adopted the "Guidelines for Medical and Health Information Sites on the Internet."

By observing such self-imposed ethics codes, and not relying on legal regulations, the role played by this system, which guarantees the independent conduct of these parties, is surely very great. However, these ethical norms do not govern independent entities. In each of the various areas efficiency can be heightened by mutually achieving the defined norms, and a course can be set to finally ensure benefit to users.

With the aim of creating an environment for the safe and effective use of the Internet in the medical field, medical specialists, attorneys, and patient representatives in Japan assembled in 1998 to form a nonprofit citizen's organization, the Japan Internet Medical Association (JIMA) [10]. JIMA first proposed the "Information Source Guideline," which set down the following three items as minimal conditions for information providers: (1) The party providing information is to be identified. (2) A contact method for questions, such as telephone or e-mail, is to be provided. (3) Notice is to be given that the user takes personal responsibility for his or her use of the information, with the premise that the information provided is not always correct or valid.

These conditions were intended to promote the provision of the basic information minimally necessary for users and to recommend prudent use of medical information. Members who agreed to conform to this guideline and provide information would be issued an emblem and would be entitled to display the emblem on their Web site. Even though information providers must take these three basic requirements into consideration whether or not they are members, the above-mentioned study found that, of the 1147 medical-related Web sites in the directory of "YAHOO!JAPAN," 13% gave no phone number, and 24% gave no e-mail address [3].

It was expected that the ethical norms which information providers must observe would be added to the above-mentioned "Information Source Guideline" and that principles of conduct for information sources in the medical and health fields would eventually be compiled. The content would include privacy protection, preservation of dignity, responsible issuance of information, notice of the principle that users take personal responsibility for use of information, and some way of addressing problems. However, new problems were foreseen in areas where such provisions could not be applied, and it would have been difficult to adopt an all-inclusive ethical standard. The e-Health Code of Ethics [11] was similar to ethical standards being considered by JIMA, and the spirit of the code was in agreement with JIMA's basic ideas. Even if there were differences in language, law, and social environment, the problems encountered in the medical use of a worldwide information network were broadly common, and problems in the same arena were shared. Hence, participation in discussions seeking solutions to such issues was considered necessary. In December 2000, JIMA created the Japanese version of the e-Health Code of Ethics [see Appendix or http://www.jima.or.jp/trust/eHealthEthics_jp1.pdf].

This ethical code, which deals with the assured safety and benefit of users when medical and health-related information and services are provided by companies or individuals, is a rational guideline for taking ethical and just actions. It is believed that information and service providers will respect this guideline and, by achieving required conduct goals, will maintain a high-quality standard for activities within e-health. This compliance will, in turn, deliver reliability to users. The important points here are the propriety and reasonableness of goals and, at the same time, the evaluation and monitoring of whether actual conduct is in line with the goals and whether goals are actually being met [12,13].

The JIMA mark (Figure 1) displayed on the Web sites of JIMA members not only indicates the site operators' agreement with JIMA's principles of conduct, it also means that if users are dissatisfied or have opinions about the information or service provided, there is a feedback function to convey complaints or opinions to the administrators of the Web site. Information providers, by constantly receiving evaluations of their work from outside sources through such feedback, will increase the level of users' trust. This process is called the "trust program," and the JIMA mark indicates participation in this program. In the beginning, the real purpose of the JIMA mark was as a tool to increase the effectiveness of the trust program. However, there was concern that, contrary to our aim, the display of the mark would convey a high evaluation of the Web site. We now believe that by presenting the code of ethics and by making the meaning of the trust program clear, the danger inherent in this mistaken interpretation can be reduced.

**Figure 1:** JIMA Mark

### Third-party Certification

As of January 2001, a new JIMA certification function is being added to this mark. Although the identity of the entity that operates the Web site is important information, objectively guaranteeing the actual correctness of that information is difficult. Here, the issue involves ascertaining who is to guarantee the correctness of information. For each server on which a Web site is placed, an identification is issued, and a third-party certification system is being provided. One requirement is that it be a corporation, and certification is by each server. Even if a single server has multiple Web sites, each one cannot always be certified. There is also the issue of cost. At JIMA, by providing a certification function for the mark, the identity of the party is verified; to prevent misuse of the mark, certification has been made possible, not at the server level, but for each Web site.

Efforts are being made to ensure observance of this ethical code. When a conflict with this standard is perceived, opinions are graciously accepted and efforts are made toward revision. It is believed that this trust mark systematically supports the trust program. For example, JIMA members mutually conducted evaluations, and, through the presentation of their opinions, improvements were made in content in terms of more appropriate methods of expression. In such experiments, not only are member sites individually improved, but examples can also be found where application is possible for the improvement of general medical-related sites on the Internet.

### Security and Confidentiality

At present, problems of security and personal information have been the biggest issue when individuals obtain medical or health-related services from the Internet or purchase medications or health-related products. Public concern has increased with the development of e-commerce. In particular, when intangibles such as services or information are provided, personal information is obtained from users (unlike in the case of purchasing physical products). Most likely, too little caution is paid in cases where such information is used. In Japan, concern about the use of personal information is low among company employees and consumers, and it has been pointed out that measures to address this issue have been slow.

Of all the kinds of personal information, it is particularly necessary to make known the importance of protecting information related to highly confidential personal medical treatment. In the questionnaire for patients used by JIMA in the above-mentioned study [3], of 645 respondents who have used the Internet, 72% said that they "believe problems occur when personal medical treatment information leaks." Similarly, to the question, "when personal medical information is transferred over the Internet, what kind of measures do you think are needed to prevent leaks, falsification, or misuse?" 50% said that "information such as names that are specific to an individual are better not sent," 41% said that "the use of personal medical information must be regulated by law," and 34% said that "data or information should be sent encrypted."

When a study was conducted on the disclosure status of proprietor information in the 1147 aforementioned medical-related Web sites, it was found that regardless of whether or not personal information had been obtained from the other party (e.g. in an online medical consultation), none of the sites explicitly stated their policy for handling personal information in the form of a privacy statement [3]. Furthermore, with regard to "measures such as the encryption of information" for the transmission of content relating to personal illness or health in the same medical consultations, it could not be confirmed that any of the Web sites used countermeasures such as encryption. So far, there are not many opportunities for medical-related Web sites to collect personal information, but this fact shows the low level of awareness among the people involved.

A basic law on individual information protection that will establish a comprehensive framework for the protection of personal information is currently being prepared in Japan. Based on this basic law, further individual laws are being studied for each field with respect to transmission, confidence, and medical treatment. In future, with the development of electronic chart proliferation and online access, the categories that will require protection and surveillance in the medical field are expected to expand in scope. Finally, yet another security problem surfaces: As we move from the closed systems of the past to open systems connected to networks, the potential risk of security being breached is increasing. In this area, as well, a multifaceted approach in pace with the progress of technology must be devised.

### The "Medical Information Usage Guidebook"

Creating and implementing a self-imposed ethical standard for providers of information and services is like purifying water (the information) that is poured into an ocean (the Internet). After the harmful and valueless information has been removed, one can expect to find something of value. But on the user side, one must be able to distinguish between the contents of information and selectively use useful information for one's own health and benefit [14]. It is necessary for users to develop the habit of critically appraising information at all times through education and mutual learning. At times, a medical expert may be a source of support in this process. At other times, support may be found in patient and family groups [15]. Guidebooks are being provided overseas for the correct and effective use of medical and health information [16], and in December 1999, JIMA created and is now publicizing its own "Medical Information Usage Guidebook." We have compiled 10 recommendations in this guidebook, with points that are easy to understand, regarding the kinds of information a user needs and the way it should be used (see Textbox 1). A user should not believe all information relating to medical treatment or health, but should make a sober comparison and analysis and use the information prudently, under the principle of taking personal responsibility for information use.

**Guideline to the Use of Medical Information on the Internet**

* Developed by the Japan Internet Medical Association.

**Use information from sites where the information provider is clearly identified.**

If the information provider is not clearly identified, responsibility that accompanies the supply of information becomes ambiguous, and the accuracy of information provided tends to be diminished. In addition, if the information is used, no adequate support can be expected, even if trouble occurs. It is important that the name, address, and contact method for the information provider be clearly provided, and that it is possible to confirm its existence.

**Use information that is not-for-profit.**

Even if the information is supposed to represent the latest science, there is sometimes a hidden profit motive. There may be product sales or special services behind the information provided. It is very important to look with a discriminating eye for some mechanism through which someone is making profit.

**Use information that has an objectively scientific basis.**

At first glance, even if information appears to be specialized, it is necessary to exercise caution with information having arbitrary content or questionable information that seems to exceed scientific understanding. One should consider whether related medical papers or articles and test data are properly quoted and whether the information has a proper scientific basis.

**Mainly use medical information provided by public medical institutions or official research institutions.**

At public medical institutions and official research institutions, such as organizations, responsibility is taken seriously, and information provided is carefully investigated and verified by committees and numerous specialists. As a result, they are sources of information with a high level of objectivity and minimal slant. However, because individual contributions are also included, it is necessary to confirm to what extent information is official. In addition, even the private sector provides information that is known to have been objectively well investigated may be said to have high levels of reliability.

**Always use recent information.**

Advances are constant and rapid in information related to health and medicine. Even the latest information, if it is not revised, at some point becomes old information, and its use value also changes. One should always check the dates of posted information as well as their revision dates.

**Compare and study multiple information sources.**

Information on the Internet is issued by people with various positions and various ways of thinking. Even if the theme is the same, viewpoints will differ depending on the information provider's position. Rather than just use one type of specific information, it is important to read and compare different information and to select the information needed for one's own purpose.

**Recognize the principle of taking personal responsibility for the use of information.**

In using information that is provided to large numbers of unspecified persons, if by chance the user suffers some misfortune, it is difficult to charge the information provider with responsibility. Basically, "information is to be used at one's own risk." Information should be used in a sober and prudent fashion.

**When in doubt, consult with a specialist.**

Some of the medical information provided in places such as the Internet does not agree with current standard medicine, and some information is ambiguous in its scientific foundations. One might enjoy early access to the latest fruits of medicine, but there is also a danger of mishaps and damage to one's own health. Do not accept all information that is provided. Always consider risks, consult with an attending family physician or medical specialist about any doubts, and obtain appropriate advice.

**Make a sober evaluation of the results from using information.**

When information is used, the value of its content should be evaluated. In order to assess the information's content and the results of its use, it is necessary to have the composure to make a calm and fair evaluation.

**If trouble is encountered, consult a specialist.**

If some trouble or damage to health is encountered when such information is actually used, do not remain silent. Consult with a medical specialist, public consultation center, or neutral third party institution. By promptly providing information, the next incident of injury or trouble may be prevented.

## Conclusion

In the past, medical treatment was structured like a pyramid, with the physician at the top, the co-medics and paramedics supporting at the periphery, and the patient buried inside, not to be seen. In the future, the patient is expected to move to the center, while the physician (professional) and support members will surround the patient at the periphery, making up a network to jointly support the health of the patient. In the development of the Internet, medical information is not the property of specific individuals; it is, rather, for the common use of all concerned parties. Information related to medical treatment and health affects the quality of life of patients and the therapy provided by physicians to patients.

This may be the era in which decisions encompass the medical treatment and welfare standards for society. According to the "Guidelines for Medical and Health Information Sites on the Internet," access to medical information through use of the Internet can change the relationship between patient and physician, and the process of medical treatment is being changed from one of the physician authority ministering advice and treatment to one of shared decision-making between patient and physician [17]. The development of information networks in medical treatment goes beyond the restrictions of time and space, systematically connects various types of information, and spawns new communication between parties with differing vantage points. This development has the potential to change the state of medical treatment, which had become rigid because of various restrictions and lack of communication, and to dramatically heighten the quality of medicine. With respect to information, it is also expected to support patients and families who have been placed in a weak position and to convey power never before enjoyed on the receiving side of medical treatment. The empowerment of patients will, at the same time, be linked to the empowerment of those providing medical care and will result in progress in medicine as a whole. This is the very direction of e-health's development.

## Appendix 1

Note: To read these documents you may need to install Japanese fonts, see http://www.adobe.com/products/acrobat/acrrasianfontpack.html

Report "Research on the Analysis of the Current State of the Provision and Use of Health Information Provided Through a New Technology Medium [in Japanese]" [3] as  [82 kB]

## Appendix 2

Japanese translation of the Washington e-health Code of Ethics [11] as  [34 kB]
