# Supplementary material for: Internet Medical Usage in Japan: Current Situation and Issues
Source: J Med Internet Res. 2001 Mar 17;3(1):e12. doi: 10.2196/jmir.3.1.e12 (PMC1761890; doi:10.2196/jmir.3.1.e12)
Supplement: Supplementary file 1 [file jmir_v3i1e12_app1.pdf]

「インターネット上の医療情報の提供と利用の実態に関する調査研究」報告書

この研究は平成 11 年度厚生科学研究（医療技術評価総合研究事業）の「新技術媒体を利用した医療等に関する情報の提供と利用の現状分析についての研究」の分担研究として実施したものです。

分担研究者代表

辰巳治之・札幌医科大学医学部教授

研究協力者

青谷裕文・滋賀医科大学
伊藤朋子・南埼玉病院
伊藤雅彦・国際医療福祉大学臨床医学センター
岩田忠俊・岩田皮フ科
内山映子・慶應義塾大学政策メディア研究科
大平整爾・日鋼記念病院
大山博司・田島病院
小内亨・桐生厚生総合病院
鎌田弘之・岩手医科大学附属循環器医療センター
上出良一・東京慈恵会医科大学
近藤靖児・札幌医科大学医学部
西藤成雄・西藤こどもクリニック
島田久夫・島田内科
隅田さちえ・厚生堂長崎病院
高井昌彦・高井内科クリニック
高橋基文・シティクリニック
宝樹真理・たからぎ医院
中島直・東京大学医学部附属病院
中野博美・京都きづ川病院
中野昌彦・京都四条病院
西田秀造・中谷皮フ科

橋本良明・伊勢崎市民病院
花井荘太郎・国立循環器病センター
平井清・柏木診療所
水島洋・国立がんセンター研究所
三谷博明・日本インターネット医療協議会
山中昇・和歌山県立医科大学
吉原博幸・宮崎医科大学

A. 研究目的

インターネットを医療の分野で新たな情報提供手段とする動きが活発になってきている。医療や健康に関する情報は、生活に関わりが深く、高齢者社会の到来とともに、国民が質の高い健康的な暮らしをしていく上でも利用価値の高いものである。情報開示の流れに加速されて、さまざまな媒体を通じて提供される情報の量も飛躍的に増えてきているが、利用者にとっては過剰な情報が、適正な判断を誤らせたり、間違った情報利用によるトラブル発生も懸念されるようになってきている。患者・家族が医療機関を決めるにあたってどのような情報を求めているか、情報源のひとつであるインターネットをどの程度利用しているか等の実情を調査し、併せて、急速に増えてきた医療機関のホームページに記載されている情報について複数の専門医によって評価を試みた。また、医療情報提供時における情報発信主体の明示、個人情報の取り扱い方針の明示等、利用者が情報を安全適正に利用できる配慮をしているかどうかの確認調査を実施した。ここで得られた成果は、患者・家族を含めた国民一般の利用者がインターネット等の新しい電子技術媒体等を通して提供される医療、健康情報を安全、有効に利用できる環境づくりを推進し、医療情報の提供者側には、今後の望ましい情報提供のあり方を討議、検討していく資料となると考える。

B. 研究方法

(1)2000 年 1 月 19 日から 2 月中旬にかけて、医療機関に通院、または入院中の患者、及びその家族を対象に、医療機関に関する情報利用の要望、並びにインターネットでの医療情報の利用状況、個人情報の扱いに関する意識調査を行った。依頼した医療機関は、大学病院 4 、一般病院 13、診療所 12、合計 29 施設であった。アンケート用紙は施設内で、主に外来の待ち合い時間を利用して記入してもらい、医療機関経由で回収した。資料１に全質問内容を記載した。

(2) インターネットの医療機関のホームページについて、発信者の主体者情報の開示状況、並びに個人情報の扱い方針の明示状況等について調査を行った。

方法は、1999 年 11 月 21 日から 12 月 5 日までの間に、大手検索サイトである「YAHOO JAPAN」の「健康と医学」の「医学」の案内ページよりリンクでたどれる医療機関のホームページを、市販のダウンロードソフト「NetRecorder」(ザクソン R&D 社製、Ver4) を使用し、トップページより 2 階層の深さまでダウンロードし、CD 記録媒体に保存したものをオフラインで閲覧し、調査した。資料 2 に、調査項目を記載した。

(3) インターネットの医療機関のホームページで提供されている医療情報の内容と、情報の提供方法について評価を行った。方法は、上記と同じように記録した医療機関のホームページの内容について、内科、小児科、皮膚科、精神科、脳神経外科の 5 分野から全部で 516 を選び、各科の専門医複数 (全部で 20 名) が分担し、施設情報、業務案内を除く医学情報に関して、その適合性について、また一般の利用者が情報を利用した時の影響度について、さらに情報の見やすさ等について評価を行った。この１次評価において、一人の医師により「門題あり」とされたサイトについては、公正な評価を期して、同じ科の他の複数医師による評価を行った (2 次評価)。資料 3-1 に、評価項目と評価法を記載した。

C. 研究結果

(1) アンケートを依頼した医療機関 29 施設から回収された回答数は、全部で 1842 名であった。その内訳は、大学病院で 353 名、一般病院で 773 名、診療所 (クリニック)716 名であった。その集計結果を資料１ - ２に示す。回答者の平均年齢は 38.4 歳、年代別に見ると 10 代が 1.6%、 20 代が 26.5%、 30 代が 26.9%、 40 代が 15.6%、50 代が 12.6%、60 代が 8.0%、 70 代以上その他が 2.8 %、不明が 5.9 %であった。性別では男性が 40.6%、女性が 55.6 %、不明が 3.7%であった。回答者の立場は、患者が 62.4%、患者の家族が 22.4 %、その他が 9.3% 、不明が 5.9% であった。居住エリア別に見ると、北海道・東北が 22.9%、関東・信越が 3 1.4 %、北陸・東海・近畿が 30.8%、中国・四国が 9.3%、九州・沖縄は 0%、不明が 5 .5%であった。

回答の内容を見ていくと、まず「医療機関（病院や診療所）についての情報を知りたいと思うことはありませんか」（複数回答) との質問に対し、「名称、所在地、交通等に関する情報」の項目中では、「医療機関の名称」61.0%（全体数に対する割合、以下同じ)、「所在地」61.0%、「電話番号」53.6%、「交通手段」48.6%、「地図」 44.6%、「ホームページアドレス」 22.4%、「電子メールアドレス」11.6 %、「ＦＡＸ番号」 10.5%、の順に要望が高かった。また、「診療科目、人員、施設等に関する情報」の項目中では、「診療時間」 69.3 %、「診療 (休診) 日」 64.5 %、「診療科名」64.1%、「駐車場の有無」48.6%、「診療時間予約の実施の有無」 48.3%、「診療科の医師 (氏名、専門領域、経

歴等) に関する情報」42.1%、「入院設備の有無」37.7%、「差額ベッドの数とその料金」 25.0%、「病室・機能訓練室・談話室・食堂・浴室等に関する事項」23.9%、「病床数・病室数」1 8.8%、「従業員数等、勤務スタッフに関する情報」11.8%、「医療機関の同一敷地内に併設されている施設の名称」 11.5 %、「療養型病床群の有無」11.2%、「施設基準関係（緩和ケア病棟、開放型病院等）に関する事項」10.9%の順に多かった。次に、「医療の内容、提供体制に関する情報」の項目中では、「診察・診断・治療に関する情報」が 65.3% と最も高く、さらに「検査や検診の案内」45.5%、「代表的な病気についての平均的な治療費の総額」44.7%、「代表的な病気についての治療・手術等の実施数、成功率」 42.2% 、と続き、以下「救急医療の体制」 41.6%、「CT、心電図等の医療機器の有無」35.0%、「紹介をすることができる他の病院又は診療所の名称」 33.2%、「代表的な病気についての平均治療日数（通院期間）」32 .0%、「予防接種の案内」29.4%、「代表的な病気についての平均在院日数（入院期間）」26.0%、「往診、在宅医療の実施の有無」26.0%、「先端医療や薬の治験の実施の有無」25.4%、「他施設との連携体制」22.0%、「診療の質を確保するための工夫」21.8%、「医療機関の地域における役割」 10.4%の順となった。そして、「患者ケアに関する情報」の項目中では、「患者、家族への病気の説明」が全体の 74 .9%と最も高く、続いて「患者の安全への配慮」38.5%、「患者のプライバシー保護や利便性への配慮」 31.4%、「患者、家族への健康教育」 30.9%、「カルテの閲覧・コピーサービスの有無」 27.3%、「患者クレームへの対応」27.1%、「医療事故への対応」 26.4%、「看護ケアの提供状況」2 5.2%、「患者の権利尊重への取組み」24.6%、「訪問看護の実施の有無」19.9 % 、「障害がある方への対応」 19.3%、「日本語が分からない方への対応」12.5 %の順であった。また、「経営、管理等に関する情報」の項目中では、「診療にかかわる安全管理」 38.1%、「第三者機関による病院評価の結果」30.8%、「サービス改善への取組み」27.3%、「医療機関全体としての倫理、理念」26 .9 %と続き、あと「診療を支える各部門の機能」 22.3%、「医療機関全体の管理体制」 21.9%、「職員への教育・研修の状況」20.2%、「職員の能力開発や看護ケアのための環境整備」19.1%、「職員を適切に確保・配置できる人事管理」 16.1%、「看護提供における理念と組織的基盤の整備状況」13.6%、「医療機関の財務状況」8.6%の順となっていた。

続いて、前記の質問中の「診療科の医師に関する情報」に関連して、「公開してほしい医師に関する情報」(複数回答) の内容を訊いてみたが、「特に専門とする分野」が 71.4%(回答者全員に対する割合）と最も高く、続いて「診療科目」53.7%、「氏名」53.7%、「勤務先医療施設名」48.4%、「提携医療施設名」26.5%、「出身大学」20.0%、「学会認定医」17.0%、「卒後年数」15.4%、「電話番号」12.9%、「住所」12.2%、「学位」10.0%、「理解できる外国語」 6.0%の順であった。

さらに「以上のような情報はどこからの資料として公開されるとよいと思いますか」(複数回答) との質問に対しては、「市町村の刊行する資料」が 42.5% (回答者全員に対する割合)、「病院・診療所が発行する資料」が 38.9%と高く、あと「都道府県の刊行する資料」25.9%、「医師会が発行する資料」22.9%、「非営利の第三者機関が発行する資料」22.8 %、「国（厚生省）の刊行する資料」 21.1%と続き、「患者［支援］団体・個人（非医療関係者）が発行する資料」 10.1%、「その他の営利団体・民間企業等が発行する資料」5. 4%となっていた。

また、この資料が「どこで入手・閲覧できればよいと思いますか」（複数回答) に対しては、「県庁・市役所・町村の役場での閲覧」49.9%（回答者全員に対する割合）、「図書館や公民館などの身近な公共施設での閲覧」42.1%というように公共の施設で利用の要望が高くなっているが、「インターネット上に公開」が 40.3%あって、新規情報媒体に対するニーズも高いのがうかがえた。最後に「一般書店等での販売」は 27.3%であった。

次に、医療相談の利用と需要に関する質問であるが、「あなたは医療について相談したい場合、どこに相談しますか」（複数回答) との問いに対し、「かかっている医療機関の相談窓口（ケースワーカ）」69.7%(回答者全員に対する割合）が最も高く、続いて「主治医以外の医師（セカンドオピニオン）」17.2%、「インターネットでの医療相談」13.2% 、「保健所 」13.0%、「市町村の健康管理課など」10.6%、「テレビ・ラジオ・新聞・雑誌などの医療相談コーナー」 6.7%、クレジット会社・生命保険会社などが行っている医療相談サービス 2.4 % の順であった。

さらに、「医療相談を利用したい場合、次のどの手段を用いますか」（複数回答) との問いには、「直接出向いて」76.7%（回答者全員に対する割合）と対面での相談希望がかなり高いものの、「電話」 51.7% 、「インターネット」 16.5 %と、対面によらない方法を希望する割合も高かった。「ファックス」は 4.8% 、「手紙 」は 3.2%と低かった。

そして、実際に「これまでに、医療について相談したことありますか」（複数回答) との問いには、「かかっている医療機関の相談窓口（ケースワーカ）」 30.2%（回答者全員に対する割合）、「主治医以外の医師（セカンドオピニオン）」10.5%、「保健所 」5.8% 、「市町村の健康管理課など」 4.8%、「インターネットでの医療相談」4.1%、「テレビ・ラジオ・新聞・雑誌などの医療相談コーナー」 2.6%、「クレジット会社・生命保険会社などが行っている医療相談サービス」1.7%の順であった。また、その時実際に選んだ手段は、「直接

出向いて」77.5% （医療相談を利用した人に対する割合）、「電話」35.5% 、「インターネット」8.4% 、「手紙」2.1%、「ファックス」1.6% 、その他 0.9% となっていた。

続いて、医療機関における情報公開に関する質問を行った。「医療機関が提供する情報の中には、広告的効果を持つものがありますが、患者側から見ても有用な情報が含まれていることがあります。患者 (家族) であるあなたにとって、個別の医療機関に関する情報の利用価値の高さを媒体別に○をつけてお答えください」(回答は各項目１つ) との問いに対して、「新聞、雑誌、書籍等の情報」は、かなり高い 22.9%、やや高い 47.5% 、高くない 29. 6%であり、「電話帳」は、かなり高い 15.9% 、やや高い 37.6%、高くない 46.5%であり、「看板、屋外掲示板、乗り物広告など」については、かなり高い 7.5%、やや高い 37.5% 、高くない 55.0%であり、「ダイレクトメール、折り込みチラシ、対外用のパンフレット」は、かなり高い 4.6%、やや高い 23.6%、高くない 71.8%、「院内の印刷物、掲示物」は、かなり高い 25.8%、やや高い 50.1%、高くない 24.1% であったが、「インターネット (電子メールやホームページ)」については、かなり高い 14.7% 、やや高い 39.2% 、高くない 46.1% であった。また、「医療機関の広報的な情報公開によって、どのような利点が期待できると思いますか」（複数回答) との質問に対する回答は、「医療機関を選択するのに役立つ」が 65.2% (回答者全員に対する割合）と最も高く、続いて「施設案内や診療案内があれば、医療機関へ行きやすくなる」 59.7%、「的確な病気の診断、治療を受けるのに役立つ」 45.9%、「気になる症状があった時、早期診断を受けに行きやすくなる」 42.5%、「近くのかかりつけ医を見つけやすい」40.9%、「技術のすぐれた医師を見つけるのに役立つ」 37.2%、「効率よい医療で医療費が節約できる」 15.7%、「患者の大病院志向の弊害を抑えられる」 10.9%、「セカンドオピニオンを探しやすい」9.4%であった。

次にインターネット（パソコン通信も含む）の利用の有無を訊いてみたが、「利用したことがある」は全体の 35.0%で、「利用したことがない」が 61.0% 、無回答が 4.0%であった。さらに、この「利用したことがない」とした回答者のうち、「今後利用したい」が 40.7%、「利用するつもりはない」が 15.2%、「わからない」37.2%、無回答 6.9%であったが、「利用したことがある」と「今後利用したい」を合わせると、全体の 59.8%になった。なお、インターネットの利用歴は、1 年以上～3 年未満 39.1%、1 年未満 36.4%、 3 年以上～5 年未満 16.1%、5 年以上 7.1%、無回答 1.2%であった。

また「インターネットを利用したことがある」と答えた人に、「現在、インターネットで医療情報をどの程度利用されていますか」と尋ねたところ、「病気、疾患に関する専門的な医学情報を得る」の項目では、「1 週間に１～数回」

3.3%、「1 ケ月に１～数回」11.0% 、「1 年に１～数回」26.4%、「利用していない」52.1%、「無回答」7.3%であった。「健康管理など病気の予防に関する情報を得る」の項目では、「1 週間に１～数回」 1.1%、「 1 ケ月に１～数回」1 0.4%、「1 年に１～数回」18.0%、「利用していない」59.7%、「無回答」10.9 %であった。「薬に関する情報を得る」の項目では、「1 週間に１～数回」1.6% 、「1 ケ月に１～数回」 7.3%、「1 年に１～数回」17.5%、「利用していない」 64.2%、「無回答」9.5%であった。「医療機関の業務に関する案内を見る」の項目では、「1 週間に１～数回」0.8%、「1 ケ月に１～数回」 7.0%、「1 年に１～数回」 17.4%、「利用していない」65.4%、「無回答」 9.5% であった。続いて、「個々の症状などに基づく個別の医療相談・健康相談」の項目では、「 1 週間に１～数回」 1.1%、「1 ケ月に１～数回」3.9% 、「1 年に１～数回」 16. 0%、「利用していない」68.8%、「無回答」 10.2%であった。「患者どうしの情報交換」の項目では、「1 週間に１～数回」1.9%、「1 ケ月に１～数回」2.8 %、「1 年に１～数回」6.0%、「利用していない」77.8%、「無回答」11.5%であった。

続いて、インターネットを利用したことがある人に「どのような医療情報サイト (ホームページ) が役立っていますか」（複数回答) と尋ねてみたが、「診療所、クリニックにより運営されるもの」 25.7%、「大学病院、研究機関により運営されるもの」23.9%、「製薬会社等、医療系の民間企業により運営されるもの」 14.1%、「患者（支援）団体・個人 (非医療関係者) により運営されるもの」 13.3%、「国（厚生省）により運営されるもの」1 2.9%、「非営利の団体・機関等により運営されるもの」 12.6%、「市町村により運営されるもの」10 .5%、「地域中核病院により運営されるもの」10.4%、「都道府県により運営されるもの」9.0%、「医師会により運営されるもの」8.7%、「非医療系の民間企業により運営されるもの」 5.1%の順だった。また、「今後、医療・福祉面において、インターネットのどのような利用法を期待しますか」（複数回答) と質問に対しては、「病気、健康管理に関する専門的情報をもっと得たい」67.4 % と「薬の効能、副作用などに関する情報をもっと得たい」 60.2%が高く、続いて、「医師等専門家による個別の医療相談」37.4 %、「患者と医師間の意志疎通、コミュニケーションを深めたい」21.7%、「在宅での医師や医療従事者による日常生活や精神面でのサポート」19.8%、「患者どうしの交流、情報交換」18.8%、「通院治療に代わる遠隔での医療」16.0% となっていた。

最後に昨今関心の高まっている個人情報の取り扱いに関する質問を行ったが、インターネットを利用したことのある回答者に対し、「インターネット上を個人の病気に関する診断、検査等の医療情報が流れる際、個人の医療情報が不用意に漏洩されたり改竄（かいざん）されたりすることの可能性についてどう思われますか」と尋ねたところ、「可能性があることを知っている」としたのは

61.3% で、「漠然と聞いたことはあるが、よくはわからない」は 28.1%、「まったく知らない」としたのが 10.6%であった。次に「インターネット上で個人の医療情報が漏れた時のリスクはどう評価されますか」との問いに対して、「個人の医療情報が漏れて問題が起こると思う」が 71.6% 、「危険はあるとしても現実的には大きな問題にはならないと思う」が 24.2%、「危険はないと思う」としたのが 4.2%だった。また、「インターネット上で個人の医療情報の流通に際し、情報の漏洩・改竄・悪用を防ぐためにはどのような工夫が必要と考えられますか」との問いに対して、「名前など個人を特定できる情報は送らないほうがいい」とする人が 49.6% 、「個人の医療情報の扱いについて法律で規制すべきである」とする人が 40.8%、「データや情報を暗号化して送るべきである」とする人が 34.0%、その他の意見が 3.1%であった。

(2) インターネットからダウンロード、CD に保存した医療機関のホームページのコンテンツで閲覧可能な医療機関サイトは全部で 1147 あった。その内訳は、大学病院 34、一般病院 526、診療所 568、個人 13、その他 6 であった。調査項目 1 の「発信者主体の氏名の明示状況」については、トップページに明記してあるが 20.7%、トップページ以外に明記してあるが 53.2%、明記されていないが 26.1%だった。調査項目 2 の「発信者主体の住所の明示状況」については、トップページに明記してあるが 47.5%、トップページ以外に明記してあるが 41.2%、明記されていないが 11.2%だった。調査項目 3 の「問い合わせ窓口の設置状況」については、そのうち、電話番号の明示に関して、トップページに明記してあるが 48.9%、トップページ以外に明記してあるが 38.5%、明記されていないが 12.6%だった。次に FAX 番号については、トップページに明記してあるが 3 3.6%、トップページ以外に明記してあるが 28.1%、明記されていないが 38.3%であった。そして、E-mail(電子メール) に関しては、トップページに明記してあるが 58.2%、トップページ以外に明記してあるが 11.2 %、E-mail の表示でなく画面上からメッセージが送信できる形態になっているのが 6.5%あり、まったく明記されていないが 24.1%であった。調査項目 4 の「個人情報の取り扱いに関する方針の明示」については、個人情報の取り扱いに関する方針が明示されているが 0.2% であったが、これも細かく明文化されたものでなく、いわゆる Pri vacy Statement のかたちになっているものは皆無であった。調査項目 5 の「情報の暗号化等の対策」については、これはホームページ上で、たとえば医療相談などで個人情報や質問、回答の内容を送受信するときに、情報が暗号化されるなどのシステムを導入しているかどうかを見るものだが、この対策がなされているサイトは 0%であった。調査項目 6 の「サイトの真正性を示す認証シールが掲示されている」については、これは当該サイトが架空のものでなく、サイトの運営者主体が第三者によって認知されていることを示す手法のひとつであるが、この認証シールが掲示されているサイトは、5 件で 0.4 %であった。

(3) 医療機関のホームページで提供される情報について、評価の対象としたサイトの数は全部で 516 であった。科目別の内訳は、内科 257、小児科 67、皮膚科 65、精神科 91、脳神経外科 36 であった。これらを、内科 5 名、小児科 4 名、皮膚科 5 名、精神科 3 名、脳神経外科 3 名の各科専門医が分担して 1 次評価を行った。各科における 1 次評価の結果を資料 3-1 に記載した。1 次評価全体としては、情報内容の適合性の評価において、サイトで提供される医療情報の内容で、「やや問題あり」とされたのが 40 で 7.8%、「かなり問題あり」とされたのが 5 で 1%あった。その理由については、一般の人が誤って情報を利用する恐れがある、検証が不十分な情報を含んでいる、現在の標準的な医学からはずれている、内容的に偏っている、記載事項に誤りがある、の順に多かったが、個別のコメントもあげられた。
情報利用者への影響度評価においては、提供される医療情報を一般市民や患者・家族が利用した場合、利用者の健康管理や疾病の予防管理においてどのような影響を与えると考えるかと尋ねたのに対し、よい影響を与えるとしたのが 43.6%、悪い影響を与えるとしたのが 1.7%、どちらとも言えないが 54.1%であった。
次に情報の提供方法の評価においては、ホームページのレイアウト、デザイン、情報の提供方法については、利用者の立場からの評価を尋ねたが、優れているが 23.1%、普通であるが 69.6%、わかりにくいが 5.8%であった。1 次評価において、内容面で、「やや問題あり」、または「かなり問題あり」とされたサイトについては、評価者による違いを考慮し、さらに同じ科の他の二人以上の医師が同様に評価する 2 次評価を行った、その結果を資料 3-2 に記載した。それによると、2 人以上の評価者によって、「やや問題あり」、または「かなり問題あり」とされたのは、内科が 5、小児科が 5、皮膚科が 13、精神科が 8、脳神経外科が 5、合計で 36 であった。割合でみると、「やや問題あり」が 1 次評価全体の 5.8%、「かなり問題あり」が 1.2%であった。参考に、１次評価、2 次評価において、「問題あり」の指摘が最も高かった皮膚科と、反対に指摘の少なかった内科における評価概括を資料 3-3 に記載した。

D. 考察

インターネットのような新規電子技術媒体を医療、並びに医療情報の提供・利用の目的に応用していく上での課題を把握するため、今回は実際に医療の受手である患者、家族において情報入手のニーズが高いと考えられる医療機関に関する情報で、どのような情報を得たいとしているかを知る必要があった。医療法における広告規制緩和に関わる問題であるが、患者、家族が自分にあった医療を求めて医療機関を決めることができるようになるには、医療機関に関する情報の提供が前提となってくる。また、その情報提供の方法も、さまざまあるが、患者はより簡便で幅広く得られる手段を望んでいることがうかがえる。イ

ンターネットは、その点において、すぐれた情報提供手段であるが、まだ現段階では普及の度合いが低く、利用者の年齢や環境によって利用度に大きな開きがあることが問題である。また、機器を有していてもどこで、どのようにうまく情報を得るかその方法がわからないという声もあった。一律に提供するのでなく、個々の利用者にあった情報提供と利用の仕方が工夫されてもいいだろう。今後とも利用者の細かいニーズをとらえていく必要がある。インターネットにおいて医療情報を提供する場合、利用者が情報利用に際して、適正な判断をなせるよう情報発信者は、発信主体者である氏名、所在地等の自己情報をすすんで提供すべきであると考えるが、実際の開示状況は十分とは言い難い。特に、双方向性が利点であるインターネットにおいて、E-mail の記載がないのは、自らその特性をふさいでいるように思われるが、情報発信に伴うトラブルも存在し、自己情報を開示しにくい事情があるかも知れない。また、精神科等の一部診療科においては、簡便な E-mail による医療相談が実際の治療を混乱させるケースも指摘され、E-mail を記載することの是非に悩む医療機関もあった。
次に個人情報の扱いについて、他領域においても関心が高まっている折り、医療情報利用の分野における個人情報の取り扱いに関する現状を調査したが、ホームページ上での個人情報の取り扱いについて、プライバシーやセキュリティへの配慮がうかがえるサイトはほとんど見当たらなかった。医療機関の患者向けのアンケート調査でも、個人情報の漏洩に対する心配が高いこととあわせて、この方面での早急な対策が望まれる。

医療機関が開設するホームページで提供されている情報の中で、主に医学情報について、複数の専門医による評価を行ったが、信頼されるべき医療機関といえども一般市民や患者・家族が利用した場合に、問題が生じる可能性のあることが示唆された。今回の評価作業は、定まった評価基準がなく複数の医師の意見を元に行われたが、評価者の設定、評価手法に試行的なところがあった。今後、医療機関としての情報提供がいかにあるべきか客観的な指標や、利用者からの意見の集成が必要かと思われた。医療技術は絶えず変化し、またそれに対しさまざまな評価法が存在するため、情報を絶対的に評価する基準は定めにくいが、インターネットような通信媒体を通じ伝搬される情報が利用者に及ぼす影響は軽視しえず、社会的に流通する医療情報を評価する仕組みが検討されてもよいと考える。また、ネガティブな側面を評価するだけでなく、優れた情報提供をしているサイトを積極的に評価し、より有用な情報提供を促すモチベーションづくりを検討する価値があろう。

E.結論

新しい情報通信インフラであるインターネット上における医療情報の提供・利用の現状を調査するため医療機関を利用する患者・家族を対象としたアンケー

ト調査を行うとともに、医療機関のホームページにおける情報提供者の自己情報の開示状況、個人情報の取り扱いに関する配慮の状況を調査し、またホームページに掲示されている情報についての評価を行った。その結果、患者・家族においては医療機関や病気や治療法に関する医学情報へのニーズが高いことが確認されたが、提供される情報の内容、またその情報の提供方法、利用法については、検討改善すべき問題があることも示された。社会全体の情報化が加速する中で、生活の質の向上に欠かせない健康や医療に関する情報が果たす役割は大きくなってきている。社会的資源として医療情報を、利用者が安全、有効に利用していくための今後の環境づくりが期待される。今日、普及の著しいインターネットを身近な医療情報の提供・利用の手段としてうまく機能させていくには、情報の提供者、利用者という相互の立場の理解の上にたった当事者レベルでの創意、工夫が求められよう。

資料 1

医療情報の利用に関するアンケート調査結果

(1) アンケートを依頼した医療機関 29 施設から回収された回答数は、全部で 1842 名だった。その内訳は、大学病院で 353 名、一般病院で 773 名、診療所 (クリニック)716 名であった。その集計結果を資料１ - ２に示す。回答者の平均年齢は 38.4 歳、年代別に見ると 10 代が 1.6%、 20 代が 26.5%、 30 代が 26.9%、40 代が 15.6%、50 代が 12.6%、60 代が 8.0%、70 代以上その他が 2.8 %、不明が 5.9%であった。性別では男性が 40.6%、女性が 55.6%、不明が 3.7%であった。回答者の立場は、患者が 62.4%、患者の家族が 22.4%、その他が 9.3%、不明が 5.9%であった。居住エリア別に見ると、北海道・東北が 22.9 %、関東・信越が 31.4 %、北陸・東海・近畿が 30.8%、中国・四国が 9.3%、九州・沖縄は 0%、不明が 5.5%であった。依頼した医療機関は、大学病院 4、一般病院 13、診療所 12、合計 29 であった。

・アンケート実施医療機関施設数　29

(内訳)	大学病院	4
	一般病院	13
	診療所	12

・アンケート実施期間　平成 12 年 1 月 19 日～2 月 20 日
・アンケート回収人数　1,842

・回答者の立場	患者	1,149 (62.4%)
	患者の家族	412 (22.4%)
	その他	172 (9.3%)
	不明	109 (5.9%)

・回答者の平均年齢　38.4 歳

・回答者の性別

1.男　748 名 (40.6 %)　2.女　1,025 名 (55.6 %)　3.不明 69 名 (3.7 %)

・居住エリア	北海道・東北	422 (22.9%)
	関東・信越	578 (31.4%)
	北陸・東海・近畿	568 (30.8%)
	中国・四国	172 (9.3%)
	九州・沖縄	0 (0%)

問１．医療機関（病院や診療所）についての情報を知りたいと思うことはありませんか。（複数回答。回答者全員に対する割合）

(名称、所在地、交通等に関する情報)

１．医療機関の名称	61.0%
２．所在地	61.0%
３．電話番号	53.6%
４．ＦＡＸ番号	10.5%
５．電子メールアドレス	11.6%
６．インターネットのホームページアドレス	22.4%
７．交通手段	48.6%
８．地図	44.6%

(診療科目、人員、施設等に関する情報)

９．診療科名	64.1%

10．診療 (休診) 日	64.5%
11．診療時間	69.3%
12．診療時間予約の実施の有無	48.3%
13．診療科の医師 (氏名、専門領域、経歴等) に関する情報	42.1%
14．従業員数等、勤務スタッフに関する情報	11.8%
15．入院設備の有無	37.7%
16．病床数・病室数	18.8%
17．病室・機能訓練室・談話室・食堂・浴室等に関する事項	23.9%
18．医療機関の同一敷地内に併設されている施設の名称	11.5%
19．療養型病床群の有無	11.2%
20．施設基準関係（緩和ケア病棟、開放型病院等）に関する事項	10.9%
21．差額ベッドの数とその料金	25.0%
22．駐車場の有無	48.6%

(医療の内容、提供体制に関する情報)

23．診察・診断・治療に関する情報	65.3%
24．検査や検診の案内	45.5%
25．予防接種の案内	29.4%
26．CT、心電図等の医療機器の有無	35.0%
27．代表的な病気についての平均治療日数（通院期間）	32.0%
28．代表的な病気についての平均在院日数（入院期間）	26.0%
29．代表的な病気についての平均的な治療費の総額	44.7%
30．代表的な病気についての治療・手術等の実施数、成功率	42.2%
31．診療の質を確保するための工夫	21.8%
32．先端医療や薬の治験の実施の有無	25.4%
33．医療機関の地域における役割	10.4%
34．他施設との連携体制	22.0%
35．紹介をすることができる他の病院又は診療所の名称	33.2%
36．救急医療の体制	41.6%
37．往診、在宅医療の実施の有無	26.0%

(患者ケアに関する情報)

38．患者、家族への病気の説明	74.9%
39．患者、家族への健康教育	30.9%
40．患者の安全への配慮	38.5%
41．看護ケアの提供状況	25.2%

42．訪問看護の実施の有無　19.9%
43．医療事故への対応　26.4%
44．障害がある方への対応　19.3%
45．日本語が分からない方への対応　12.5%
46．カルテの閲覧・コピーサービスの有無　27.3%
47．患者のプライバシー保護や利便性への配慮　31.4%
48．患者クレームへの対応　27.1%
49．患者の権利尊重への取組み　24.6%

(経営、管理等に関する情報)

50．医療機関全体としての倫理、理念　26.9%
51．第三者機関による病院評価の結果　30.8%
52．診療を支える各部門の機能　22.3%
53．診療にかかわる安全管理　38.1%
54．医療機関全体の管理体制　21.9%
55．職員への教育・研修の状況　20.2%
56．看護提供における理念と組織的基盤の整備状況　13.6%
57．職員の能力開発や看護ケアのための環境整備　19.1%
58．サービス改善への取組み　27.3%
59．職員を適切に確保・配置できる人事管理　16.1%
60．医療機関の財務状況　8.6%
61．その他　0.6%

〇かかっているところがあるので特に必要ない
〇看護婦さんの数（特に夜勤）
〇決まっているので特にない
〇子供の長期入院 (3 ヶ月) の体験から、医者の人間としての資質を問いたい
〇アンケート項目すべてに関する実態
〇託児
〇地域医療への状況説明
〇患者を大事に扱ってくれる為言葉態度に医者をしてきくばりをしてほしい→患者はモルモットではないし感情のある人間なのです

問２．公開してほしい医師に関する情報があれば、その内容を選び、番号に○をつけてください。（複数回答。回答者全員に対する割合）

１．氏名　53.7%
２．勤務先医療施設名　48.4%

３．	住所	12.2%
４．	電話番号	12.9%
５．	診療科目	53.7%
６．	特に専門とする分野	71.4%
７．	学会認定医	17.0%
８．	提携医療施設名	26.5%
９．	出身大学	20.0%
10．	学位	10.0%
11．	卒後年数	15.4%
12．	理解できる外国語	6.0%
13．	その他	1.3%

○診療経歴、年齢、経験、実績
○医師の評価
○人柄、性格
○①過去に勤務した病院名②人間性
○医療（治療）に対する理念
○勤務先の住所は知りたい、自宅は不要
○診療方針
○専門医としての格付け
○得意とする技術
○論文等

問３．以上のような情報はどこからの資料として公開されるとよいと思いますか。（複数回答。回答者全員に対する割合)

１．	市町村の刊行する資料	42.5%
２．	都道府県の刊行する資料	25.9%
３．	国（厚生省）の刊行する資料	21.1%
４．	病院・診療所が発行する資料	38.9%
５．	医師会が発行する資料	22.9%
６．	その他の営利団体・民間企業等が発行する資料	5.4%
７．	非営利の第三者機関が発行する資料	22.8%
８．	患者［支援］団体・個人（非医療関係者）が発行する資料	10.1%
９．	その他	0.8%

○インタ-ネットのホ-ムペ-ジ
○病院、診療所
○信用できる調査会社
○書店での書籍として
○電話帳

〇どこでもよい
〇インターネット、従ってホームページの開設
〇営利に関係なく医師の人格、技能、倫理に対して確実な判断をなされたものであって欲しい。
〇交流会
〇特になし

問４．その資料はどこで入手・閲覧できればよいと思いますか。
（複数回答。回答者全員に対する割合）

１．県庁・市役所・町村の役場での閲覧	49.9%
２．図書館や公民館などの身近な公共施設での閲覧	42.1%
３．一般書店等での販売	27.3%
４．インターネット上に公開	40.3%
５．その他	2.7%

〇病院・診療所など医療機関
〇電話・FAX 等で情報が得られればよいと思う
〇タウンペ-ジ等
〇地域（自治体等）の公報にて
〇圏内の医師であれば市報などで配布
〇回覧板などより身近なもの
〇該当する施設自体で
〇各医療機関での待ち時間を利用してのインターネット閲覧
〇各家庭に配布 (地域ごと）または郵送
〇学校
〇新聞
〇図書館
〇医師会 or 保健所
〇アンケートに記載のすべての場所
〇道で発行する刊行物でもよし
〇わからない、特になし

問５．あなたは医療について相談したい場合、どこに相談しますか。
（複数回答。回答者全員に対する割合）

１．かかっている医療機関の相談窓口（ケースワーカ）	69.7%
２．主治医以外の医師（セカンドオピニオン）	17.2%
３．保健所	13.0%
４．市町村の健康管理課など	10.6%

５．テレビ・ラジオ・新聞・雑誌などの医療相談コーナー　6.7%
６．インターネットでの医療相談　13.2%
７．クレジット会社・生命保険会社などが行っている医療相談サービス　2.4%
８．その他　5.2%
〇主治医、知合い（友人・親戚）の医師・医療関係者
〇知人、親戚、同僚、友人
〇会社の健康管理室、診療所
〇病院、医療機関、薬局
〇どこに相談したらよいかわからない
〇看護婦
〇家族
〇相談したことがない
〇医大ならば主治医、家庭医学百科
〇近所の元保健婦さん
〇保健所は全く頼りにならない（単なる公務員にすぎない）
〇医療者間の情報
〇特になし

問６．あなたは医療相談を利用したい場合、次のどの手段を用いますか。
（複数回答。回答者全員に対する割合）

１．直接出向いて　76.7%
２．電話　51.7%
３．ファックス　4.8%
４．手紙　3.2%
５．インターネット　16.5%
６．その他　0.3%
〇友人・知人からの紹介
〇公開された医師の中から選出して相談が可能であれば利用したい
〇医療書

問７．あなたはこれまでに、医療について相談したことありますか。もしあれば、利用したものの番号に〇をつけてください。（複数回答。回答者全員に対する割合）
なければ、問９．へ

１．かかっている医療機関の相談窓口（ケースワーカ）　30.2%
２．主治医以外の医師（セカンドオピニオン）　10.5%
３．保健所　5.8%

４．市町村の健康管理課など　4.8%
５．テレビ・ラジオ・新聞・雑誌などの医療相談コーナー　2.6%
６．インターネットでの医療相談　4.1%
７．クレジット会社・生命保険会社などが行っている医療相談サービス　1.7%
８．その他　4.2%
○主治医、知合い（友人・親戚）の医師・医療関係者
○知人、親戚、同僚、友人
○会社の健康管理室、診療所、保険組合
○病院、医療機関、薬局
○家族
○人間ドック
○ＴＥＬ悩み相談
○医師会
○運動指導者、栄養士
○刑務協済医療相談
○市販の本（病院リスト）を見て
○第三者機関の代表
○電話での相談センター、知人等
○訪問看護婦
○医療雑誌の記者
○***医大病院の精神科の入院患者にケースワーカが必要である

問８．あなたが問７の医療相談を利用したとき、次のどの手段を用いましたか。(複数回答。医療相談を利用したことのある方 [問 7 のいずれにも○をつけなかった人数 966 人から計算した] に対する割合）

１．直接出向いて　77.5%
２．電話　35.5%
３．ファックス　1.6%
４．手紙　2.1%
５．インターネット　8.4%
６．その他　0.9%
○検診時

問９．医療機関が提供する情報の中には、広告的効果を持つものがありますが、患者側から見ても有用な情報が含まれていることがあります。患者 (家族) であるあなたにとって、個別の医療機関に関する情報の利用価値の高さを媒体別に番号に○をつけてお答えください。（回答は各項目１つ。各項目の回答者に対

する割合）

・新聞、雑誌、書籍等の情報

　１．かなり高い　22.9%　　２．やや高い　　47.5%　　３．高くない　29.6%

・電話帳

　１．かなり高い　15.9%　　２．やや高い　　37.6%　　３．高くない　46.5%

・看板、屋外掲示板、乗り物広告など

　１．かなり高い　 7.5%　　２．やや高い　　37.5%　　３．高くない　 55.0%

・ダイレクトメール、折り込みチラシ、対外用のパンフレット

　１．かなり高い　 4.6%　　２．やや高い　　23.6%　　３．高くない　 71.8%

・院内の印刷物、掲示物

　１．かなり高い　25.8%　　２．やや高い　　50.1%　　３．高くない　24.1%

・インターネット (電子メールやホームページ)

　１．かなり高い　14.7%　　２．やや高い　　39.2%　　３．高くない　46.1%

　その他
　○近所の評判（口コミ）、友人・知人からの情報・評価
　○近所の医院
　○ラジオ等
　○ＴＶなどニュース番組などに先生が出たりするとすごいと思ったりする
　○患者さんがつくってるＨＰ
　○行ってみないとわからない
　○市の発行物
　○信頼にたるか否の判断基準とならない
　○主治医の人脈
　○わからない、利用したことがない

問１０．医療機関の広報的な情報公開によって、どのような利点が期待できる

と思いますか。（複数回答。回答者全員に対する割合）

１．	施設案内や診療案内があれば、医療機関へ行きやすくなる。	59.7%
２．	医療機関を選択するのに役立つ。	65.2%
３．	技術のすぐれた医師を見つけるのに役立つ。	37.2%
４．	的確な病気の診断、治療を受けるのに役立つ。	45.9%
５．	近くのかかりつけ医を見つけやすい。	40.9%
６．	セカンドオピニオンを探しやすい。	9.4%
７．	気になる症状があった時、早期診断を受けに行きやすくなる。	42.5%
８．	効率よい医療で医療費が節約できる。	15.7%
９．	患者の大病院志向の弊害を抑えられる。	10.9%
10．	その他	0.2%

○医療不祥事の防止につながる
○遠出している時に急病等になっても、すぐ病院がさがせるようになる
○自分に合う先生を探せる
○自分の生活に合ったものを選択できる

問１１．あなたはインターネット（パソコン通信も含む）を利用していますか。
（回答は１つ。回答者全員に対する割合)

１．	利用したことがある。	35.0%
２．	利用したことはない。	61.0%
３．	無回答	4.0%

問１２．あなたはインターネットを今後利用したいですか。（回答は１つ。問１１で「利用したことはない」と答えた人に対する割合)

１．	利用したい。	40.7%
２．	利用するつもりはない。	15.2%
３．	わからない。	37.2%
４．	無回答	6.9%

問１３．あなたのインターネット利用歴はどれくらいですか。
（回答は１つ。問１１で「インターネットを利用したことがある」と答えた人に対する割合）

１．	１年未満	36.4%

２．１年以上〜3 年未満　39.1%
３．３年以上〜5 年未満　16.1%
４．５年以上　7.1%
５．無回答　1.2%

問１４．あなたは現在、インターネットで医療情報をどの程度利用されていますか。（回答は各項目１つ。問１１で「インターネットを利用したことがある」と答えた人に対する割合）

病気、疾患に関する専門的な医学情報を得る。
１．１週間に１〜数回　3.3%
２．１ケ月に１〜数回　11.0%
３．１年に１〜数回　26.4%
４．利用していない　52.1%
５．無回答　7.3%

健康管理など病気の予防に関する情報を得る
１．１週間に１〜数回　1.1%
２．１ケ月に１〜数回　10.4%
３．１年に１〜数回　18.0%
４．利用していない　59.7%
５．無回答　10.9%

薬に関する情報を得る
１．１週間に１〜数回　1.6%
２．１ケ月に１〜数回　7.3%
３．１年に１〜数回　17.5%
４．利用していない　64.2%
５．無回答　9.5%

医療機関の業務に関する案内を見る
１．１週間に１〜数回　0.8%
２．１ケ月に１〜数回　7.0%
３．１年に１〜数回　17.4%
４．利用していない　65.4%
５．無回答　9.5%

個々の症状などに基づく個別の医療相談・健康相談
１．１週間に１〜数回　1.1%

２．１ケ月に１～数回　3.9%
３．１年に１～数回　16.0%
４．利用していない　68.8%
５．無回答　10.2%

患者どうしの情報交換
１．１週間に１～数回　1.9%
２．１ケ月に１～数回　2.8%
３．１年に１～数回　6.0%
４．利用していない　77.8%
５．無回答　11.5%

その他
〇現在は利用していない
〇インターネットで医療情報を入手したことなはい。→方法が分からない。
〇今まで利用した事はないが、今後情報を検索して、利用価値があれば多いに利用したい
〇医療関係では不利用
〇ＭＬＴの情報収集
〇患者の出しているＨＰで通信
〇急な耳の痛みでインターネットで調べ坐薬を入れました
〇仕事の関係で感染症や食中毒についてのデータは見る事がある
〇自分では、まだ進んで出来ないので、教えてもらい又は打ち出している
〇主人がインターネットの会社を設立したので
〇地図（病院、診療所の場所を確認

問１５．どのような医療情報サイト (ホームページ) が役立っていますか。（複数回答。問１１で「インターネットを利用したことがある」と答えた人に対する割合）

１．市町村により運営されるもの	10.5%
２．都道府県により運営されるもの	9.0%
３．国（厚生省）により運営されるもの	12.9%
４．大学病院、研究機関により運営されるもの	23.9%
５．地域中核病院により運営されるもの	10.4%
６．診療所、クリニックにより運営されるもの	25.7%
７．医師会により運営されるもの	8.7%
８．製薬会社等、医療系の民間企業により運営されるもの	14.1%
９．非医療系の民間企業により運営されるもの	5.1%

１０．非営利の団体・機関等により運営されるもの 12.6%
１１．患者（支援）団体・個人 (非医療関係者) により運営されるもの 13.3%
１２．その他 4.2%
○見ていない、利用したことがない
○医療関係では、見ていない、利用したことがない
○どのようなサイトがあるのかわからない、アドレスがわからない
○わからない
○保健所
○医師個人？のＨＰ
○会社のホームページ
○特になし

問１６．今後、医療・福祉面において、インターネットのどのような利用法を期待しますか。（複数回答。問１１で「インターネットを利用したことがある」と答えた人に対する割合)

１．病気、健康管理に関する専門的情報をもっと得たい。 67.4%
２．薬の効能、副作用などに関する情報をもっと得たい。 60.2%
３．在宅での医師や医療従事者による日常生活や精神面でのサポート。 19.8%
４．通院治療に代わる遠隔での医療 (保険適用、非適用いずれも)。 16.0%
５．医師等専門家による個別の医療相談。 37.4%
６．患者と医師間の意志疎通、コミュニケーションを深めたい。 21.7%
７．患者どうしの交流、情報交換。 18.8%
８．その他 2.3%
○医療施設の情報照会、得意分野、設備等
○期待しない、利用するつもりはない
○BSlloh のような医療教育相談
○病院の自己ＰＲ
○医療面では利用していないのでわからない
○あまり医療機関へ行って治療を受けたりしないので当分必用ない
○インターネットなんかに頼らず外来で来た時にきちんと病状を説明できる医者が少ない
○特になし
○わからない

問１７．インターネット上を個人の病気に関する診断、検査等の医療情報が流れる際、個人の医療情報が不用意に漏洩されたり改竄（かいざん）されたりすることの可能性についてどう思われますか。（回答は１つ。問１１で「インターネットを利用したことがある」と答えた人に対する割合)

１．可能性があることを知っている。 61.3%
２．漠然と聞いたことはあるが、よくはわからない。 28.1%
３．まったく知らない。 10.6%

問１８．インターネット上で個人の医療情報が漏れた時のリスクはどう評価されますか。（回答は１つ。問１１で「インターネットを利用したことがある」と答えた人に対する割合)

１．個人の医療情報が漏れて問題が起こると思う。 71.6%
２．危険はあるとしても現実的には大きな問題にはならないと思う。 24.2%
３．危険はないと思う。 4.2%

問１９．インターネット上で個人の医療情報の流通に際し、情報の漏洩・改竄・悪用を防ぐためにはどのような工夫が必要と考えられますか。
（複数回答。問１１で「インターネットを利用したことがある」と答えた人に対する割合)

１．名前など個人を特定できる情報は送らないほうがいい。 49.6%
２．データや情報を暗号化して送るべきである。 34.0%
３．個人の医療情報の扱いについて法律で規制すべきである。 40.8%
４．その他 3.1%
○監視システム
○情報を扱う人の意識を高める
○本人しかアクセスできない方法を徹底する
○その HP 上に患者専用の ID、PASS で入ることのできるページを作ればよいと思う。(メンバーページのようなもの)
○今のところは防ぐのは困難
○いわゆるハッカー等が侵入した場合に警報がでるとともに、履歴が判る等の工夫をしてほしい
○インターネットを使わない
○日本人はもっと個人情報の大切さを学校教育から知るべき。その為にも③で少しは歯止めをかけるべき。
○基本的に防ぐことは出来ないと思うから防ぐ為ではなく、起きたらどう対処するかを考えるべき
○流れるのは途中ではなく、病院等の末端である。（暗号化は行われるが末端では意味をなさない。）
○上記の事が心配なので自分自身ではインターネットでの利用はしない様にしているため、わかりません。

○各自の責任を問う事をユーザーに自覚させる事の徹底化
○問 18 の回答で健康食品販売当に情報がもれると困ると注釈あり
○ブライバシー情報の活用は、世の中で抵抗もあり、ルール、ガイドラインを真剣に取り組む必要があると思われる。暗号化などの技術的な要素も必要だが、セキュリティという観点ではその運用が最も主要な 要素の１つと思われる。いずれにしろ個人的にはインターネットの共用は、方向性としてまちがっていると思います。がんばって下さい。
○特に対策なしでいいと思います
○しない
○わからない

資料 2

医療機関ホームページにおける情報発信主体者評価結果

・評価対象サイト数 1147

(内訳)	大学病院	34
	一般病院	526
	診療所 (クリニック)	568
	個人 (主に医療関係者によるもの)	13
	その他	6

(1) 情報発信者主体の明示状況に関する調査

ホームページにて医療情報を発信する主体者 (以下、発信者主体と略する) に関する情報がどの程度明示されているかについて調査した。

(2) 発信者主体の氏名の明示状況

発信者主体である個人または法人、団体名が明示されているかどうかを調べた。病院名が一般に知られていても、Web の管理者又はコンテンツの責任者と思われる人の名前が掲示してなければ、「明記されていない」と判断した。

1.トップページに明記してある	20.7%
2.トップページ以外に明記してある	53.2%
3.明記されていない (閲覧できた階層の範囲内で)	26.1%

(3) 発信者主体の住所の明示状況

発信者主体である個人または法人、団体の住所が明示されているかどうかを調べた。

1.トップページに明記してある	47.5%
2.トップページ以外に明記してある	41.2%
3.明記されていない (閲覧できた階層の範囲内で)	11.2%

(4) 問い合わせ窓口の設置状況

情報の利用に際して、利用者が発信者主体に問い合わせ等の必要の生じた時のために問い合わせ窓口が明示されているかどうかを調べた。

・電話番号の記載

1.トップページに明記してある	48.9%
2.トップページ以外に明記してある	38.5%
3.明記されていない (閲覧できた階層の範囲内で)	12.6%

・FAX 番号の記載

1.トップページに明記してある	33.6%
2.トップページ以外に明記してある	28.1%
3.明記されていない (閲覧できた階層の範囲内で)	38.3%

・E-mail の記載

1.トップページに明記してある	58.2%
2.トップページ以外に明記してある	11.2%
3.明記されていない (閲覧できた階層の範囲内で)	24.1%
4.フォーム送信ページがある	6.5%

(5) 個人情報の取り扱いに関する方針の明示

ホームページのオンライン上で個人情報を送受信する際、プライバシー保護に関して、情報受信側において個人情報の取り扱いに関する方針 (いわゆる個人

情報保護のための行動指針、Privacy Statement) がわかりやすく明文化されて掲示されているかどうかを確認した。

1.個人情報の取り扱いに関する方針が明示されている	0.2%
2.特に明示されていない	99.8%

(6) 情報の暗号化等の対策

ホームページのオンライン上で医療相談や問い合わせなどで、利用者が個人情報を送信する時、送信情報の暗号化などの対策がとられているかどうかを確認した。

1.対策がなされている	0%
2.特に対策はなされていない	100%

(7) Web 認証シールの有無

サイトの真正性を保証する何らかの方策がとられているかを判断した。第三者機関による認証シールがあるが、システム的に証明機関との間で「なりすまし」を防ぐ機能が確保されている高度のレベルのものから、リンクにより照合できる簡易レベルのものも含めて「あり」とした。

1.サイトの真正性を示す認証シールの類いが掲示されている	0.4%
2.特に掲示されていない	99.6%

資料 3-1

医療機関のホームページにおいて提供される医療情報の内容評価結果 (１次評価)

・評価対象サイト数　516

(内訳)	内科	257
	小児科	67
	皮膚科	65

精神科	91
脳神経外科	36

・評価方法

評価対象としたサイトを各科目別に各科専門医 (構成は、内科 5 名、小児科 4 名、皮膚科 5 名、精神科 3 名、脳神経外科 3 名) が分担してそれぞれ 1 次評価を行った。

質問 1　当該サイトで提供されている医療情報の内容はどう評価されますか？ (%は全体数 516 に対する割合)

1 内容的に問題はない	471 (91.3%)
2 一部に不適切と思われる個所があり、やや問題がある	40 (7.8%)
3 不適切と思われる個所が多くあり、かなり問題がある	5 (1.0%)

「やや (若しくは、かなり) 問題がある」とされた場合、その理由は何でしょうか？ (複数回答)

(1) 現在の標準的な医学解釈からはずれている	9
(2) 検証が不十分な情報を含んでいる	14
(3) 内容的に偏っている	9
(4) 記載事項に誤りがある	1
(5) 一般の人が誤って情報を利用する恐れがある	16

その他、自由コメントとして以下の指摘があった。

＜内科＞

○医療情報についてはリンクの形で他のサイトの情報に依存している。したがって、そのサイトの情報によっては問題になる可能性がある。
○最近の花粉症の増加の原因についての記述が偏っており、参考文献なども紹介されていない。星状神経節ブロックには副作用は認められず、安全な治療法との記載があるが、この施設では今までそうであったかも知れないが，一般的にはどうか。
○「***町『***検診』での当院の二次検査で早期がんを含む有所見が数例発見され、早期治療を開始致しました。」との記述で、母数が不明。過去の成績を出すのならば、正確な数字を出すべき。「当院内科は***医科大学***教室の***

教授の協力を得て、検査・診断から治療まで、看護婦及び医療技術者とともに最新医療機器を駆使し、高度先端医療及び慢性疾患管理を徹底いたしております。」との記述で、医療設備の具体的内容が不明。最新医療設備という言葉はあいまい。また、病院の性格は概要が記載されているが、定量的につかみにくい。病床数が明らかではない。
○まだ未完成なＨＰ。
○工事中が多すぎて情報とは言えない。もっと完全な状態で公開すべき。
○「骨密度想定装置→測定」のケアレスミス。
○「ＭＲＡを用いると、脳主要血管の閉塞・狭窄を容易に観察することができ、クモ膜下出血の原因となる脳動脈瘤（直径５ミリ以上）を検出できるので、脳卒中の予防に効果があります。」とあるが、予防に効果があるという確証はないのではないか。

＜小児科＞

○いわゆる健康器具を使用した治療室の宣伝と思われる。
○ホームページ上に、連絡先、所在地が見あたらない。
医療機関なのか、個人のページなのかわからない。提供内容も整理されていず、教科書から引き写しの印象のあるところがある。一般に利用される医療情報としては不適切で混乱を生じる可能性がある。

＜皮膚科＞

○手、足のほくろはできるだけ早くとると記載されているが、必ずしもそうではない。
○随所に独自に開発した外用剤があることが記載されているが内容は明らかでない。
○自由診療の料金を他院と比較している。
○化粧品の宣伝等、医療機関のページとしてはいかがなものかと思います。
○ケミカルピーリング以外の脱毛や美容形成の料金記載がない。
○全体として医療より脱毛、植毛などの美容に誘導している。医療情報の内容も毛髪関係は妥当であるが、美肌関係は一部内容に乏しい。
○本来泌尿器科医であるためか皮膚疾患と一部の性感染症の記載が不十分であり、また一部正確性を欠くところがある。
○レーザー脱毛で足が細くなる、半導体レーザーで「シミ」が薄くなる、生理痛が軽快したなど問題ある記載が散見される。患者の経験談を掲載して治療の妥当性を予想させる方法は医療機関としては好ましくない。
○治療前後の症例写真の表示はよいが、そのとおりになる保証はない。

＜精神科＞

○Neulorogy を神経科と訳している。
○現状では積極的に行わない方向性に変化してきている強制入院制度に関して、積極的に行うべきである印象で紹介がなされている部分が多々ある。また、思春期の心性について分析しつつも結局は隔離入院制度を導入している風に取れる箇所がある。
○高校生の息子の相談例で簡単に診断を決めつけてしまい不安をあおる恐れがある。

＜脳神経外科＞

○内容が余りに少なく、評価のしようが無い。
○医師から見れば適切と思われるが、一般人には難しいのでは。

質問２　当該サイトで提供される医療情報について、これを一般市民や患者・家族が利用した場合、情報利用者の健康管理や疾病の予防管理においてどのような影響を与えると考えられますか。(%は全体数 516 に対する割合)

1.よい影響を与える	225 (43.6%)
2.悪い影響を与える	9 (1.7%)
3.どちらとも言えない	279 (54.1%)
4.無回答	3 (0.6%)

上記で 1 または 2 と回答し、「悪い影響を与える」とされた場合、それは具体的にどのような影響かとの問いの対して、自由コメントとして以下の指摘があった。

＜皮膚科＞

○水道水に塩素が入っていなければアトピー性皮膚炎にならないかのような錯覚を与えている。
○ステロイド外用のありもしない副作用を強調し恐怖感をあたえ、自らの治療法でアトピーが治癒するとうたってアトピービジネスである。
○間違った解釈により症状が悪化する可能性もあり。
○十分に検討されたとはいえない論文を掲載し、患者を誘導している。
治療前、治療後の症例写真を多数掲載しているが、果たして提唱する治療法だけが軽快理由か不明。自家製の製品を購入するよう誘導している。価格も記載されていない。

○美容的処置を求める利用者については最低限の情報が提供されているが、疾病を持つ患者に関してはどこまでが保険診療扱いしてもらえるか情報が十分とはいえない。
○レーザーの効果を過大に評価している。また起こりうる副作用、効果の持続、費用などが明示されていない。レーザー治療の体験談などを掲載しているが、医療機関としての品性が疑われる。
○保険診療と自費診療に関し何ら記載がない。自費診療の際の費用の記載がない。

＜精神科＞

○母親が周囲に責められたり、必要以上に自責的となり、抑うつ的になる恐れがある。

質問３　当該サイトで提供される医療情報について、ホームページのレイアウト、デザイン、情報の提供方法等について、情報利用者の立場からみた評価を聞かせてください。(%は全体数 516 に対する割合)

1.優れている　119 (23.1%)
2.普通である　359 (69.6%)
3.わかりにくい　30 (　5.8%)
4.無回答　8 (　1.6%)

その他、自由コメントとして以下の指摘があった。

＜内科＞

○デザインはまあまあだが、組織図から各科の案内にリンクするが、診療科からは行けないなど、柔軟性に欠ける。
○非常に丁寧で、見る人のニーズを考えた作りで、感心させられる。
○わかりにくい。ＨＰの肝心な部分（診療案内、診療科紹介、受診される方へ）が録画されてないためかどうか、一般の人が見て満足できる内容ではない。***大のイントラネットの公開みたいで、インターネット・イントラネットをマニアックに運用しているところが目に付いた。
○非常に良く計画されて作成されたＨＰ。
○文字が見にくい、背景もＷＩＮまたはＭＳ-office の基本的なものを流用しており洗練されていない。
○最初から構成をよく練られて、施設全体の参加が感じられる作り。病院ＨＰのお手本、医師の参加が不足しているのが残念。

○HP のデザインはシンプルだが、カラーコーディネイトのためか非常に見やすい。
○ピロリ菌のリンク先が形成外科になっている、整合性がない。

＜精神科＞

○診療時間が表示されていない。
○ＢＧＭがうるさい。
○各職種、施設の説明がわかりやすい。
○診療内容がないのは少し問題あり。また、日本温泉気候物理学会認定療法医とは、どんなものなのか。一般的な資格でない気がするが。
○適応障害の説明が、あまりにも単純に、母親が問題、と決めつけている印象を与える。
○アルコール依存症専門医のエッセイが好印象。
○相談コーナーがあるのがよい、全体的にわかりやすい。

質問４ 当該サイトについて他にご感想、ご意見があれば記入ください、との問いの対して、自由コメントとして以下の指摘があった。

＜内科＞

○病院の紹介が中心
○医療，福祉関係の行政についての解説が多いが，一部政治的色彩が強い。
○情報不足、病院案内が中心と思われる。
○褥そうの情報は充実している。
○一部に有用な情報がある、ただし病院の宣伝が主体。
○胃のはなし、スポーツについての話題がある。
○養生訓は参考になる。
○病院案内のみ
○診療内容が開示されている。一部医療関係者向けの情報有り。
○教科書的な内容，リンクが充実している。
○「かわら版」はおもしろい。成人病関連の情報が豊富。
○健康一口メモがある。
○テレメディシンによる遠隔医療の研究の情報がある。
○病院案内中心，薬局で聞くわかりづらい言葉がでている。
○慢性肝炎の説明あり。
○介護，リハビリの相談コーナーがある。
○インターネット診察室あり。
○各科に簡単な医療情報がある。

○診療内容、治療成績の説明がある。内容が専門的。
○多汗症 HP がある。
○トピックスの医療情報が詳しい。
○肛門疾患の情報あり。
○心疾患，糖尿病の情報あり、やや古い。
○個人的な意見が掲載されている。
○医学雑談は個人的な意見。
○わかりやすい内容でいい。
○漢方治療についての内容なのでよくわからない部分が多い。
○総合的には優れていると思われる。メンバーに麻酔科の先生がいれば、
星状神経節ブロックの副作用について確認してほしい。
○利用者にわかりやすいＨＰを目指しているようで非常に親切である。
○医療技術者の参加が感じられない、単なる病院案内。
○内容的には、かなり具体的に診療内容を紹介して、また人間ドックの申し込みがＨＰから可能にするなど意欲的だが、ＨＰの構成が複雑で直感的にわかりにくい。
○それぞれの頁を異なる担当者が作り、それを統合して調整する係がいないために頁をめくるたびに違和感を生じさせる作り。内容的には単なる病院紹介で、問題無いが、わかりにくい。
○ＨＰは余分な複雑さを出さないシンプルな構成でわかりやすい。内容も深くは踏み込んでいないが、ホスピスの紹介など、このＨＰをみた人に影響を与えると思われる。
○診療案内が録画されて無く、一般の患者向けの姿勢を見ることができなかった。それ以外は、ほとんどが医療関係者向けの内容となっている。
○単なる病院の紹介。啓蒙としては堅すぎる内容の広報。
○あまり深く掘り下げてはいないが、丁寧で親切な診療案内、医療内容の紹介が好感。ＨＰも凝ってはいなく、わかりやすい。
○構成がよく練られており、ややくだけた感もあるが好感もてる。病院ＨＰのお手本。啓蒙も程よく入っている。
○構成がよく練られており、画面も明るく、癌の話が中心であるのに好感もてる。病院ＨＰのお手本。
○安心感の伝わる落ち着いた構成、病院ＨＰのお手本。
○医療従事者にアピールする内容。
○臨床試験第一相のボランティア募集をしている。
○腎不全・透析の情報多い。
○施設紹介型 HP、移転予定の工事現場の写真がユニーク。
○薬の説明から、給食部門など多岐にわたる内容になっている。
○施設・職員紹介型であるが、健康祭りなどの地域連携に力を入れているのが伺える HP。

○施設紹介型 HP。看護学生のページがユニーク。
○施設紹介型の HP、東洋医学センターの紹介あり。
○友の会組織になど、地域向けサービス多し。
○病診連携室と看護教育の紹介が充実。施設紹介型。
○施設紹介型の HP、IVR 等の治療の説明多し。
○放射線技師が中心となって作成したものらしい、放射線障害に多くの記載あり。
○圧倒的な量のコンテンツ、職員のエッセーが面白い。
○施設紹介のデザインが優れる。
○施設紹介型の HP、透析に力を入れているのが伺える。
○疾患の説明から、地域連携の講演会、日帰り手術センターの紹介など内容の充実した HP である。
○社会派の理事長のコラム記事の量に圧倒される、医療というよりも福祉サービスの紹介が多いのが特徴。
○一般人向けに抗がん剤の解説とあざの解説は充実している。また、研修医向けのコース紹介と病診連携を意識した内容も充実している。
○掲示板が立ち上がっている。また趣味の車コーナーがなど医療というより趣味の HP らしい。
○生活習慣病対策や病気の解説のボリュームは圧巻。
○腎不全の解説に力いれている。
○甲状腺疾患とカウンセリングについて詳細な内容あり。
○病気の説明、診療内容の説明等にととまらず食事療法の解説も充実している。
○理事長の趣味の絵画等があるのが特色、施設紹介型 HP。
○施設紹介型 HP、政治的主張もあり。
○循環器病の術後の患者の FAQ が充実している。
○施設紹介型の内容が多いが、地域連携および福祉サービスの紹介もある。
○含まれる情報量そのものが少ない。
○病院の紹介中心でありその他の健康情報の発信は少ない。
○医療情報量は豊富であるが、内容に検証不足のこともあり、影響としてはプラスマイナスであり相殺されている。
○病院情報主体のサイトであるが、小児科のサイトに社会性があった。
○病院情報が主体で患者に必要な医療情報に乏しい。
○病院内情報にすぐれているがその情報提供に限られている。
○病院臭さのないｈｐであるが、提示される内容は乏しい。
○患者の必要としている医療情報に乏しいが、看護婦関連の情報は多い。
○豊富な医療情報が掲載されている。
○麻酔関係の情報が豊富であるが、他の部門は乏しい。
○一般市民に必要な情報は乏しく、探しにくい。
○医局のホームページでもあり、医療情報に乏しい。

○皮膚科の医療相談のコーナーがあるが、実体は不明。
○古い情報があり、その量も限られている。
○病院内情報が主であるが医療相談室がある。
○画像を多用した重たいｈｐであった。医療相談室があった。
○医局中心のサイトであり一般患者になじみにくい内容である。
○医療情報に少し古いところがあるが、病院情報以外の医療情報も比較的豊富である。
○提供される情報量はわかりやすいが少ない。
○小児科部門の医療情報が比較的豊富である。
○病院情報以外の医療情報に乏しい、相談室は開店休業状態。
○若手医師により立ち上げられたサイトで豊富な医療情報がある。
○院内の診療情報は詳しいが、それ以外の医療情報は乏しい。

＜小児科＞

○小児科以外の科のページは外来表だけである。小児科のページは非常に親切。
ただし、感染症流行状況が更新されていないのは不都合。
○このサイトは小児科医など専門家むけに作られている。専門家向けとしては
すぐれている。一般向けではない。
○shockwave などを利用して凝った作りになっている。利用者によってはかえってとまどう場面があるかもしれない。
○有用な情報が記事として含まれているが、どこにあるのか検索しにくい。
○一種の広告の域を出ない。医院の所在地や機能も医療情報ではあるが。
○病院の機能をかなり詳細に紹介している。主に医療者向けの情報提供と考えられるが、来院・入院される患者さんにとっては見て安心できる情報であるかもしれない。
○外来担当表などの基本的情報のみ。
○簡潔に有用な情報をまとめている。
○内容は簡潔かつ有用。情報利用に関する注意書きまで掲示され、極めて良く考えられた内容である。
○自院の医療内容を宣伝に堕さずによく伝えている。地域の医療機関情報として有用。
○患者、医療者両方にとって有用な情報源。非常に幅広く、表現方法も美しい。
○内容に良い部分があるが、情報提供として整理されていない印象が強い。
○dead link が多い。
○患者さん向けの情報提供が豊富だが、一部医療者向けのような込み入った
内容になっている。一般向けの情報としては整理できていない印象がある。
○禁煙に対する積極的なアピールが素晴らしい。
○ホームページを開設されている先生の考えなどを読んでいるとなるほどと思

うことが記載されていて面白い。ページもさほどこったものではないが、適度に軽い画像でイラストが使われており、見やすさと見た目の良さを適度に両立させている。
○受診された方やサイトをご覧になった方からの「提案箱」を準備されているのは大変よいと思う。
○コンテンツを作ったのは誰なのか記載が無い、イニシャルだけのようである。
○トップページで小児科と産婦人科のページに分かれる。産婦人科までみたが、こちらはほとんどが工事中。サイトにどんなコンテンツが格納されているか一目で分かるように、インデックスページは工夫されている。
○地図が分かりやすい、親しみあるイラストがある。
○このサイトの最大の魅力のコンテンツは同院が発行する月刊院内報であり、トップからリンクを張り訪問者にもっとアピールするべきだろう。
○アンケートのフォームや感染症情報のメール新聞の発行など見るべきところもある。担当医がどういった学会に所属し認定医・専門医の評価を得ているのかなど記載されている事も評価したい。

＜皮膚科＞

○警告を促すべきサイトである。
○宣伝だけである。
○「やけど」専門の医療機関である。自費診療のみ取り扱っているとのことである。秘伝の軟膏でどんなやけどでも手術なしで治すことをうたい文句にしている。現在の医学常識では、第 3 度熱傷は植皮の適応であり、保存的治療では瘢痕治癒になるので可能なかぎり手術をすることになっている。
○医学的な記載はほとんどない。
○宣伝であり医療情報に乏しい。
○植物や昆虫の写真と解説がわかりやすく、有益である。
○単なる広告である。
○力作である。利用価値高し。
○院長の経歴や、得意分野などが明示されていると、患者の役に立つと思う。予約診療の有無などが明示されているとよいと思う。
○自費診療で扱われる疾患の解説が主なので、一般の方の健康管理にはあまり役立たないかもしれません。ただし、このように診療内容を絞って具体的に解説がなされている点は評価できる。
○某医療関係の掲示板でこの医院を誹謗する書き込みを見たことがある。
○レーザー脱毛やケミカルピーリングの話題が主であり、一般市民に良い影響を与えるのかそうでないのか評価しづらい。
○一部に深海水の使用など医学的に検証が不十分な内容もあるが、全体的に見ると大きな問題はないので、総合的に「問題なし」と判断した。

○一人の医師で皮膚科も内科も外科も診療が出来るのかどうかの方が、ホームページの内容よりも気になりますが。
○国立**病院への交通機関の案内のみのホームページだった。
○業者が作成しているようでどの程度情報を提供してくれるか未知である。
○内容がやや専門的で一般の人には理解しにくと思う。
○医院の情報だけで医学的な情報は記載されていない。
○アトピー性皮膚炎の治療にステロイドを使用しないと宣言されているが、自らの経験に基づいて主張されていることで医学的にはこういう意見があっても問題ないと思う。
○common skin diseases について大変分かりやすく書かれている。皮膚ガンについての記載がほとんどないのは、それなりの配慮の結果と解釈する。このサイトの妥当性を損ねるものではない。医者のかかり方についてもご本人の経験を生かした記載で一般的心理レベルの人が読めば理解できる内容である。サイト内のキーワード検索ができるのも親切である。患者からの画像付き質問メール受付も本当に機能しているなら、すばらしいことである。全体としてご本人の温かい人柄がにじみ出ているサイトである。リンクも妥当である。
○アトピー性皮膚炎の治療において、患者との信頼関係が大切であるとか、一般的注意事項は妥当なものであるが、十分に検討されたとは言い難い学説 (学会発表) を根拠に自己の治療法の妥当性を説き、また自家製品を販売してそれに誘導している点は問題がある。
○本サイトの評価は保険診療主体の皮膚科としてではなく、自費を前提とした美容が主体の診療所として評価した。なお、***病院皮膚科診療部長を顧問にしていることを顔写真入りで掲載しているのは、***病院からみていかがなものか。
○本サイトの評価は保険診療主体の皮膚科としてではなく、自費を前提とした美容が主体の診療所として評価した。院長の著書を利用したレーザー脱毛の宣伝は最近の流行である。レーザー脱毛の料金が記載されていることは評価できるが果たして妥当なものか疑問である。レーザー脱毛に関する情報提供が乏しい。特に再発に関して記載がない。著書を読めと言うことか。また、植毛に関してはカウンセリングを勧めるのみで料金記載がない。
○本サイトの評価は保険診療主体の皮膚科としてではなく、自費を前提とした美容が主体の診療所として評価した。わきが、レーザー脱毛、コラーゲン注射など美容的色彩が強いが、それぞれに対する治療法の原理、適応、実際の処置、得失、起こりうる後遺症、治療効果の持続、費用などが詳しく記載されており、美容関係のサイトを評価する際の標準となろう。
○多数の皮膚疾患について FAQ が詳細に掲載されており、患者に役立つ情報が満載である。オンラインでアドバイスも行っているようで、機能していれば評価できる。
○多数の皮膚疾患について一般的医療情報が網羅されているので役立つであろ

う。
○泌尿器科専門医だけあって包茎の手術法やパイプカットの詳細解説や術後経過、費用が明記されていることは評価される。
○本サイトの評価は保険診療主体の皮膚科としてではなく、自費を前提とした美容が主体の診療所として評価した。院長はレーザー治療を長年手がけているが、一部のレーザー治療について、その効果を十分な検証のないまま過大に表現している．また保険適応のある疾患に対するレーザー治療がどの程度保険で行われているか不明である。院長の出た雑誌記事などを掲載しているのは、医療機関としては不適切である。更にアンケートに答えた人に院長の著書を送るというのも、医療機関の情報提供として疑問が残る。
○本サイトの評価は保険診療主体の皮膚科としてではなく、自費を前提とした美容が主体の診療所として評価した。自費診療するのであればそれなりの情報を提供すべきである。

＜精神科＞

○うつ病につき、当該患者の心の持ち様や家族の対応に力点が置かれすぎ。治療の必要性、有効性にもう少し力点を置くべき。
○センターの紹介を中心にし、医療情報は抑えられている。
○研究所・所長の業績紹介が多く、医療情報が極めて少ない。
○相談事例を問答式にまとめている点はわかりやすいが、受診させることを目的としているような印象がある。
○施設と制度の紹介のみで医療情報はなかった。
○疾病に関する説明は簡潔明瞭である。
○デイスペースの紹介に終始し、医療情報はない。
○アルコール依存症に力点を置き、他の疾患の紹介はごく少ない。
○制度、診療内容について丁寧に説明してある。
○施設の紹介のみである。
○外来スケジュールの紹介だけであった。
○若干だが医療情報が掲載されていた。
○施設・スタッフの紹介だけであった。
○アルコール依存症についての相談を受け付けているのは優れている。
○公開可能な相談のみに回答しているのは独創的。
○内容は充実しているが、ページが多かった．また使用されている写真に写っている方々の同意が得られているのかどうかが気になった。
○精神科独自の公費負担制度などが紹介されていて良い。
○院内報の抜粋が掲載されており読み応えはあるが、文章だけで十分に適切な内容が伝え切れているかどうかの検証が不十分に思える箇所がある。問題と言うほどではないが。

○患者さんと思われる方の顔が写っている写真を公開しているが，本人の了承を取ったのかが気になる。
○患者さんの顔がしっかりと隠してあって十分に配慮されていると思われる。
○入院形態，入院必要品について詳しい．病院までのバスの時間が掲載され便利。
○最低限度の病院紹介内容で，好感が持てる。
○患者さん本人に許可を受けて写真を掲載しているかが気がかり。
○全体に清潔なイメージのデザインがされている。
○平易に書かれている分、若干厳密性に欠けるところがある。場合によっては誤解を招くこともあり得る。
○医療情報の提供を目的としたホームページではない。
○医療情報はほとんどない。
○情報としては非常に乏しい。外面的なことのみ。
○施設紹介に重点を置いている。
○基本的に施設紹介。福祉制度の解説がプラスの情報として利用できる可能性がある。
○医療情報の提供という点からいうと、余談があまりに多く雑多すぎて不親切である。また、登校拒否の記載のところは他の項目に比すと簡略すぎ、医療を必要とするケースも一面的なように思われる。見解の相違であるとも言える。
○本サイトの評価には、提唱されている「ピクノジェノール」という健康食品への評価が必要になる。一般的な医学的見解から言えば、健康食品に高度のものを期待するのは問題がある、ということになろう。但し見解の相違だと言ってしまえばそれまでである。質問箱は丁寧に答えていて、評価すべきものがある。
○「やさしい精神科の話」の項が、平易に書かれている。一部の記載に議論があり得るものもあるが、全体としては一般向けの情報として評価できるものがある。

＜脳神経外科＞

○ほとんど医療情報がない。単に病院の案内だけであり、広告に終始しているといって極言ではないようだ。もう少しなんらかの医療情報が欲しい。
○ややドグマチックであるが、全般的には優れている。一般の人には難しすぎる点もあるがじっくり読めばためになるものと思われる。
○病院の簡単な紹介だけで内容がなく医学的な情報がまったくない。
○一般の人むけというよりはすでに診断された患者さんをかかえた家族向けというニュアンスが強い。そういう感覚で読めば良いサイトである。医療情報の説明がやや専門的という感じである。

○医療情報に関しては優れていてしかもわかりやすい。説明としては非常に良い。ただ医療情報がどこにあるのか一見しただけではわからないようになっている。むしろ隠しているかのような印象を与える。非常に残念である。
○医療情報は全くない。たんなる病院紹介である。
○あまり宣伝くさくもなくまずまずのサイトである。記載が良心的であるのも好感が持てる。
○内容、レイアウトとも非常に充実している。
○ほとんど病院の広告であって医療情報についてはあまりページも割かれておらず見るべきものは無い。
○国立病院としては異例のことであるが表紙に院長の名前が大きく出ている。内容は広告そのもので医療情報がほとんどない。まるで私立病院のようなサイトである。
○脳神経外科的治療が必要な疾患でも保存的に治療できるものもあるが、すべて手術治療しか方法がないような記載が見られる。もっと神経内科からみた記載も必要である。
○文章は平易で分かりやすい反面、具体的な表現に乏しい感じがする。外来当番表に役職名の表示は必要ではないか。教育施設のホームページとしては、ページスタイルが全くのプレインテキスト形式で、もう少しデザインがあってもいいのではないか。一般の人に対するものと、医師に対するものが一部混在している。***分院に関する説明が少ないのではないか。mailto 無し。
○施設紹介が主であり、一般の人の医学的要求に対する説明は無い。取りあえず作成した表札のような感じのページである。
○ページスタイル、デザインともに良い。内容の説明も詳細で丁寧である。敢えて言うなら、医学内容の説明に関して、テキスト段落が大きいので、もう少しクラスター化した方が見やすいのではないか。関係機関のリンクを増やして、是非利用価値を高めて欲しい。よく行き届いて秀逸な出来あがりと言える。
○簡潔の極みで、いわゆる表札のみである。おそらく管理者が他施設に赴任などのためと思われる。リンク先の病院は大学関連施設であるが、プロバイダー提供の無料のページなのはどうかと考える。mailto 無し。
○施設紹介、関係者論文紹介、医局員紹介などは申し分無いと考える。一般の人の医学的要求に答える部分が無い。その他にも一般の人の利用を考えると、今一つ検討が必要か。
○産経新聞連載の「脳を守る」は、膨大な量の掲載をしておられ、一般の人の利用にも大変に有用と考える。全体としては医師向けの感じである。敢えていえば、ページスタイルが簡潔で、もう少しデザインがあってもいいのではないか。
○脳神経関連・脳卒中専門病院としての病院紹介ページである。全体としては施設紹介が主のページである。一般の人の医学的要求に対する部分は少ない

感じである。
○脳神経外科専門施設としての病院紹介ページである。施設紹介のみで、一般の人の医学的要求に対する部分は無し。
○分かりにくい領域の医学的内容を詳しく丁寧に、そしてきわめて分かりやすく説明されており、制作者の人柄が感じられる。
○地域の脳神経外科の拠点としての、施設紹介ページである。***生活習慣病シリーズの説明は、一般の人にも分かりやすくて有用である。
○施設紹介が多いが、医学的内容は画像も取り入れて良い。
○地域の脳神経外科の拠点としての病院ページである。施設紹介が主であり、医学的内容の説明が少ない。ご意見コーナーを設けて、読者にレスポンスしている。
○地域の脳神経専門施設としての病院紹介ページである。ページスタイルは丁寧な作りである。
○疾患や治療に対しての詳細はなし。公的刊行物よりの資料は有益。
○単なるチラシ。
○患者・利用者の声が載っているが、一般人はこういう情報を好むだろう。もちろんこれが偏向した内容という危険性もある。　疾患については自身のホームページでは触れず、他の HP を利用している。これがいいのか悪いのかは現在のところわからない。
○単なるチラシ、副院長の著書の広告。
○患者とのメールのやり取りを載せている。内容的にはまずまずの模範的回答。
○単なる病院案内、疾患についての説明なし。
○***大とは銘打ってあるが、個人の HP、まずまずの出来、しかし一般人にも理解できる内容かどうかは疑問である。
○個人の HP、医学的内容はまずまず。
○内容は余りにもシンプルすぎて、誤解を生ずる恐れがある。
○放射線技師が中心となって作成したものらしい、放射線障害に多くの記載あり。

資料 3-2

医療機関のホームページにおいて提供される医療情報の内容評価結果 (2 次評価)

・評価対象サイト数　　45

(内訳)

内科　　10

小児科	5
皮膚科	16
精神科	8
脳神経外科	6

・評価方法

1 次評価で、項目１の「情報内容の適合性評価」において、各科分担評価した結果、担当の評価者から、「一部に不適切と思われる個所があり、やや問題がある」若しくは「不適切と思われる個所が多くあり、かなり問題がある」と指摘があったものについて、同じ科目の他の評価者が複数名で再評価を行った。

・最終結果

各科における 2 次評価を含めた最終の評価結果は以下のとおりだった。

＜内科＞ 全体数 257

二人以上の評価者によって「やや問題あり」とされたサイト数	5 (1.9%)
二人以上の評価者によって「かなり問題あり」とされたサイト数	0 (0%)
２次評価によって、「問題あり」とされなかったサイト数	5 (1.9%)
問題なしのサイト数	247 (96.1%)

＜小児科＞ 全体数 67

二人以上の評価者によって「やや問題あり」とされたサイト数	5 (7.5%)
二人以上の評価者によって「かなり問題あり」とされたサイト数	0 (0%)
２次評価によって、「問題あり」とされなかったサイト数	0 (0%)
問題なしのサイト数	62 (92.5%)

＜皮膚科＞ 全体数 65

二人以上の評価者によって「やや問題あり」とされたサイト数	8 (1 2.3%)
二人以上の評価者によって「かなり問題あり」とされたサイト数	5 (7.7%)
２次評価によって、「問題あり」とされなかったサイト数	3 (4.6%)
問題なしのサイト数	49 (75.4%)

＜精神科＞ 全体数 91

二人以上の評価者によって「やや問題あり」とされたサイト数	8 (8.8%)
二人以上の評価者によって「かなり問題あり」とされたサイト数	0 (0%)
２次評価によって、「問題あり」とされなかったサイト数	0 (0%)
問題なしのサイト数	83 (91.2%)

＜脳神経外科＞ 全体数 36

二人以上の評価者によって「やや問題あり」とされたサイト数	4 (11.1%)
二人以上の評価者によって「かなり問題あり」とされたサイト数	1 (2.8%)
２次評価によって、「問題あり」とされなかったサイト数	1 (2.8%)
問題なしのサイト数	30 (83.3%)

＜総合＞ 全体数 516

二人以上の評価者によって「やや問題あり」とされたサイト数	30 (5.8%)
二人以上の評価者によって「かなり問題あり」とされたサイト数	6 (1.2%)
２次評価によって、「問題あり」とされなかったサイト数	9 (1.7%)
問題なしのサイト数	471 (91.3%)

資料 3-3

医療機関のホームページで提供される医療情報の内容評価の概括

１次評価、２次評価において、「問題あり」の指摘が最も高かった皮膚科及び「問題あり」の指摘が最も少なかった内科における評価概括を以下に行ってみた。

＜皮膚科＞

医療機関のサイトに掲載されている情報の分類としては、１）疾病の原因、治療など医学的知識に関する情報 、２）その医療機関で受けられる診療についての情報に大きく分類できる。また、医療機関については、診療形態から見ると、１）保険診療主体の皮膚科、 ２）自費主体で、特殊な理論、療法を掲げる医療機関、 ３）自費主体の整容的治療を行っている医療機関（美容外科、形成外科、皮膚科を併せて標榜）に分類できる。ホームページで提供されている医学知識に関する情報については、保険診療を行っている皮膚科単独のサイトで提供さ

れているものは、医学界で一般的に認められている医学情報をインターネットで広く提供する点で、内容には問題はないと考えられる。また、整容治療を行っている医療機関でも、たとえばレーザー治療の原理や効果、後遺症などについての情報が詳細に掲載されている場合には問題も少ないが、そうした情報なしに、有利な効果、作用だけがとりあげられているケースは、情報利用者に誤解を招く恐れがある。今後はより高度で詳細な evidence 付きの情報が公的機関から発信されることが望ましいだろう。

また、特殊理論・療法を掲げるサイトにおけるその内容は、明らかに現在の医療常識を逸脱するものがあり、これは評価者によらず共通して不適切情報としてあげられた。一番判定に悩むのは、例えばアトピー性皮膚炎でステロイドを使うか使わないか、という問題を典型例として、学会でも病気の解釈や治療法に議論が分かれる場合である。特殊理論・療法との区別は評価者によっても個人差が出る可能性があるが、提供される情報を、ひとつの見解、情報として見ておいてもいいサイト、というランクが必要かも知れない。次に、診療内容に関する情報については、保険診療主体の皮膚科では診療内容についての情報もほとんど問題点はない。むしろ診療案内的な情報については、もっと利用者のニーズに応えられるよう、積極的に提供されてもいいだろう。保険診療主体でなく、自費を主体とする特殊理論・療法を行っている医療機関では、時に治療費が不案内で、行われる治療の医学的妥当性に疑問が残るケースもあった。

特に、整容的治療を行う医療機関では、自由診療であっても医療である以上、治療費、治療の効果、起こりうる後遺症など、医学的常識、社会的通念から見て提供すべき情報が必要でそれらを満たしているかをどうかを評価することも重要になってこよう。自費であるため掲載されていることと、実際の治療内容に大きな隔たりがないかは、治療を受けてみないと確認できない。例えば、保険診療の適応となるいくつかのあざの治療が自費なのか保険診療を適応してもらえるのかどうか、受診してみないと分からないのは問題であろう。治療費のディスカウント広告や院長の著書による広告、特定の化粧品購入の誘導などの問題点を持つサイトもあった。

＜内科＞

内科のサイトはその提供している情報の内容により、１）施設情報のみのサイト、施設情報の他に、２）医学的な情報を提供するサイト、３）医学的情報以外の情報を提供するサイト、４）積極的にインターネットを利用しているサイトに分類できる。今回内科に振り分けられたサイトの多くは１）に分類されるものであった。

まず、施設情報に関しては、大部分が診療内容、診療時間、所在地などの情報であり、問題はなかった。しかし、中には先進医療機器、先端医療、といったあいまいな表現を使用し、具体的な内容について説明のないものがあった。

医学的な情報については、その内容が「問題あり」とされたサイトは５サイトと他科のサイトに比べて少なかった。その内には、ペインクリニックに関する情報で問題ありとされたものが２サイト、椎間板ヘルニアの治療に関する情報で問題ありとされた１サイト含まれている。したがって、厳密には内科領域に関する情報で「問題あり」とされたサイトは２つだけであった。総じて、内科についての情報の内容については問題ないと考えられる。これは、内科診療の大部分が保健診療であり診療方針が共通していること、病院のサイトが多くひとつのサイトでも複数の人により情報提供されているためと思われる。しかしながら、医学的な情報のレベルをみた場合、平易なものからかなり専門的なものまで幅広かった。「瓦版」、「・・新聞」、「一口メモ」などと対象が一般向けであることが見て取れるものがある一方、医療関係者向けの情報と思われるものが混在しているケースもあった。一般向けか、医療関係者向けかなど、どのような人を対象にした情報であるかをはっきり明記するような工夫が必要であろう。また、医学的情報を提供する病院の中には、その病院の専門性をうちだし、特色ある医学情報を提供しているサイトがいくつか見受けられた。その他、手術成績などを積極的に開示している病院もあった。このような情報は今後患者側が病院を選択する上で重要なものとなるかもしれない。今後のインターネットの利用方法のひとつとして注目されるものである。しかし、このような情報が普及するにつれ、都合のよい情報のみを公開するなど、問題が出てくる可能性も考えておかなければならない。

医学的情報以外の内容として、福祉行政の解説や、地域医療との連携についての情報など有用な情報を提供するものがあった。また、病院のイベント紹介や、スタッフのエッセーを載せるなど、医療周辺の雰囲気を伝えることをねらいとしたページもあった。

さらに、インターネットの積極的な利用として、双方向性という特長を生かし、メールによる医療相談の受付、あるいはインターネットを通じた人間ドックの申し込み等の受付を行うサイトがあった。

情報の提供方法については、サイトの見やすさホームページの構成、デザイン、配色などについてはそれぞれのサイトなりの工夫がみられた。リンク方法や構成などがよく練られているサイトがある一方、まだ未完成なまま公開してしまっているサイトもあるなど仕上げのレベルも千差万別であった。なお、今回内

科として分類されたサイトはサーチエンジンである YAHOO に準処したものになっていた。その結果、内科に分類されたホームページには総合病院等の大規模な病院が多く含まれ、診療所の比率が少なかった。このことが今回の評価の結果に影響を与えたかも知れないことを付記しておく。

(終)
