# Supplementary material for: Internet Medical Usage in Japan: Current Situation and Issues
Source: J Med Internet Res. 2001 Mar 17;3(1):e12. doi: 10.2196/jmir.3.1.e12 (PMC1761890; doi:10.2196/jmir.3.1.e12)
Supplement: Supplementary file 2 [file jmir_v3i1e12_app2.pdf]

2000年5月18日制定　　(11/18update)

eHealth 倫理コード

制定の趣旨

「eHealth 倫理コード」の目的は、世界の人々が自身の健康を管理したり、また医療従事者が患者の健康を管理したりする際に、考えられるリスクを十分に理解した上で、インターネットの持っている可能性を実現していくところにある。

はじめに

インターネットは、人々が医療情報やヘルスケアを提供したり、受けたりする方法を変革しつつある。医療に関連した目的でインターネットを利用する者―すなわち、患者や医療従事者、行政担当者、研究者、医療製品の製造販売業者、医療サービス提供者などーはすべて、一致協力してインターネットを利用するための安全な環境をつくり、ヘルスケアのニーズを応えられるよう、インターネットの価値を高めていく必要がある。

医療情報や医療製品、医療サービスは、健康を増進させるとともに、時に害を及ぼす可能性もあるため、インターネット上で医療情報を提供する組織や個人は、信頼できる情報源として質の高い情報を提供し、また利用者のプライバシーを保護するとともに、医療分野のオンラインでの商取引及びオンラインでの医療サービスの提供において、いわゆるベストプラクティスといわれる最善の行動基準を達成していく義務がある。

インターネットの医療サイト及びサービスの利用者は、利用するサイトや製品、サービスについて評価・検証を重ね、オンラインでの医療情報や製品、サービスに関する実効性あるフィードバックを行うことによって、インターネット医療の価値と有効性を確かなものにしていく責務を担うものである。

定　義

医療情報には、健康維持や疾患の予防・管理のための情報、健康・医療の関するその他の意志決定を行うための情報などが含まれる。

情報には、医療製品及び医療サービスの購入・利用を決定するための情報も含まれる。

情報はデータ、文書、音声、映像の形態をとる場合がある。

情報はプログラミングや双方向性を生かした高度の機能拡張を伴う場合がある。

医療製品には、薬剤や医療機器、診断や疾患及び傷害の治療や健康維持に用いるその他の製品が含まれる。医療製品には、米国食品医薬品局（FDA）または英国医薬品庁（MCA）などの行政当局による認可が必要な薬剤及び医療機器と、規制管理のないビタミン、ハーブ、その他栄養サプリメントなどの製品の両方が含まれる。

医療サービスには、特定の個人向けの医療ケアや助言、診療記録の管理、あるいは治療法の決定、診療費支払請求、請求書作成業務などに関して、医療提供者と患者、医療保険会社やヘルスケアプラン、医療機関などの間で行われるコミュニケーション、医療を支援するためのその他サービスなどが含まれる。

医療サービスには、メーリングリストや掲示板、チャットルームその他の医療情報の交換を目的としたオンラインの場も含まれる。

医療情報と同様、医療サービスもデータ、文書、音声、映像などの形態をとる場合があり、プログラミングや双方向性を通じた機能拡張を伴う場合がある。

医療関連目的でインターネットを利用する者は、医療情報や医療製品、医療サービスをオンラインで提供する組織および個人が以下のガイドラインに賛同することを求める権利を有する。

公正であること

①サイトの利用法、提供される製品やサービスの購入・利用に関し、もし消費者が知り得た場合、消費者の理解を

医療関連の目的でインターネットを利用する人たちは、アクセスするサイトや利用するサービスが確かで信頼できるものであることを自分で判断できることが求められる。サイトは以下の各項を明示するものとする。

**左右する可能性のあるような情報がある場合は、これを開示する。**	・ サイトの運営主体者、またサイトの運営や提供されるサービスによって明らかな経済的利益を持つと考えられるものがあれば、その個人や団体の名称<br><br>・ サイト運営またはサービス提供の目的<br>たとえば、単に教育目的であるとか、医療製品や医療サービスの販売であるとか、あるいは個別の医療ケアやアドバイスの提供であるとかの目的を明らかにする。<br><br>・ 一般に分別のある人が、サイトの提供する情報、製品、サービスに関する利用者の理解に何らかの影響を及ぼすと考えうるような関係（経済上、職務上、あるいは個人的その他の関係）があれば、その関係について<br>たとえば、そのサイトに商業上のスポンサーやパートナーがいる場合は、スポンサー／パートナーが誰であるか、またそのスポンサー／パートナーがサイトのコンテンツを提供しているかどうかをはっきりさせる。
	**誠実性**
**②真実を伝え、嘘をつかない。**	インターネット上で医療情報を探す人たちは、製品やサービスについて真実が記載され、受け取る情報が誤解を招くような方法で提供されていないことを確認できる必要がある。サイトは以下の各項について、事実をありのままに伝えるものとする。<br><br>・ 医療製品や医療サービスの販売促進のために用いられるすべてのコンテンツ<br><br>・ 製品またはサービスの効果効能、性能、有益性に関する記述<br><br>サイトは、製品やサービス、団体の宣伝もしくは販売を意図するコンテンツと、教育的、科学的コンテンツとを明確に区別するものとする。

クオリティ(質)

③正確で、理解しやすい、最新の医療情報を提供する。

インターネット利用者は医療・ヘルスケアに関して賢明な決定をするにあたり、サイトが正確かつ十分な裏付けのある情報や質の高い製品、サービスを提供していると期待する権利を有している。

提供する医療情報の正確性を期すために、eHealth サイト及びサービスの提供者は誠意をもって以下の目標の遂行に努めるものとする。

- 製品やサービスの説明に用いる情報を含め、提供される情報は厳正かつ公正に評価する。

- 現時点で入手できる最良のエビダンス(根拠)に基づく情報を提供する。

- 個別の医療ケアやアドバイスを提供する際には、有資格の医師が行うことを確実にしておく。

- 情報が科学的研究や専門家のコンセンサス、識者に基づくものか、若しくは個人の経験や見解に基づくものかどうかを明示する。

- 論争中のテーマが取り上げられていることを告知する。また、そのような場合には、公平で偏りのない立場に立って、妥当と見なせるすべての見解を紹介できるように努める。
  たとえば、前立腺癌に対する外科手術と放射線療法など、特定の疾患には治療法に選択肢があることを利用者に助言する。

情報及びサービスは、消費者にとって理解しやすく、利用しやすいものでなければならない。サイトは情報を掲示し、製品やサービスを説明するにあたって、以下の点に留意するものとする。

- 想定される利用者にとって、明解で読みやすく、適切とされる言語を用いる。
  たとえば、サイトが想定する読者の主要言語を用いて、文化的に適切な表現で記述する。

・ 利用者によって想定される可能性のある特別なニーズを採り入れた表現法を用いる。
たとえば、視覚に障害のある利用者向けに、大きなフォントやオーディオ・チャンネルを採用する。

主として教育や科学的な目的で情報を提供するサイトは、サイトのコンテンツ・エディターのみが編集コンテンツを決定し、不適切と思われる広告を拒否する権限を確保することによって、編集方針や業務の独立性を保証するものとする。

消費者には、入手する情報が最新のものであることを求める権利がある。サイトは以下の各項を明示するものとする。

・ 情報の掲示年月日（最初の情報から更新されている場合は、現在掲示されている情報のバージョン）

・ 掲示情報の最終校閲年月日

・ サイト情報の大幅な変更の有無。大幅な変更が生じた場合は、最終更新年月日。

また、サイトが提供する医療情報や医療製品、医療サービスを判断するにあたって、利用者が必要とする情報を提供する。

利用者個人は、インターネット上で得た医療情報の質を自分で判断することが求められる。サイトは、以下の点を告知することによって、コンテンツの作成過程を明瞭かつ正確に記述するものとする。

・ サイトあるいはコンテンツの提供者が利用している情報源が何であるか。参考資料や情報源へのリンクも含む。

・ サイトがどのようにコンテンツを評価しているか、またコンテンツを評価するためにいかなる基準が用いられているか。他のサイトやサービスに特定のリンクを張る際の根拠なども含まれる。
たとえば、サイトの編集委員および編集方針を記載する。

医療製品及び医療サービスが政府の規制監督下にある場合、サイトは利用者に対して、提供している製品（医薬品や医療機器など）が米国 FDA や英国 MCA などの規制当局による認可を受けているかどうかを明らかにする。

インフォームド・コンセント（リスクの開示と決定権の確保）

④個人情報の収集と、その使用もしくは供与について、利用者が決定する権利を尊重する。

医療に関連した目的でインターネットを利用する人たちは、個人情報が収集される可能性があることを告知されるとともに、自己の個人情報の収集、利用、他への供与を承諾するか否かを選択できる権利を有する。また、自らの意志で商用サービスを利用するに際し、そのタイミングや方法を選択したり、同意したり、コントロールしたりする権利を有するものとする。

サイトは以下の点を明確に開示するものとする。

・ インターネット上で利用者のプライバシーが開示されるリスクがあること。

  たとえば、誰かがあるサイトにアクセスしている時に、他の組織や個人が、当該サイトに無断で利用者の個人情報を収集できる可能性があること。また、一部の法域（欧州連合など）では、他の地域に比べてより厳重にプライバシー保護に取り組んでいること。

サイトは、利用者の明確な承諾を得ることなく、個人情報を収集、利用、供与してはならない。個人情報の提供に関し、利用者が十分な説明を受け、内容を理解した上で、決定することを確実にすべく、サイトは以下の点を明瞭かつ正確に告知するものとする。

・ サイト利用時に収集されるデータの詳細

  たとえば、サイトのどのページに利用者がアクセスしたか、あるいは利用者の氏名、E メールアドレス、利用者の健康状態やオンラインでの購買行動に関する特定の情報など。

・ データの収集者

当該サイトの運営者または第三者など。

・　収集されたデータの用途

たとえば、科学的研究の一環として、利用者によりよいサービスを提供するための一助とする、あるいは特定個人向けの医療ケアやアドバイスを提供する。

・　サイトが収集したデータを意図的に他の組織や個人に供与しているか否か。供与している場合は当該情報の詳細。

・　どのような組織や個人に、収集したデータを供与しているか、またどのような利用法を想定しているか。

たとえば、サイトが利用者の個人情報を他の組織や個人に供与する予定があるかどうか、また、その目的について。あるいはいつの時点で、自国以外の組織や個人に利用者の個人情報が供与されるか。

・　以下の目的で個人情報を収集、利用、供与する場合は、利用者の承諾を得るものとする。

たとえば、科学的研究用途や、利用者に新製品やサービスの情報を提供するなどの商業的目的でサイト閲覧者のデータを収集利用する場合や、利用者の個人情報を他の組織や個人に供与する場合など。

利用者が個人情報の提供を拒否した場合に起こりうること

たとえば、利用者の特別な要望に応じてサイトが提供している情報を個別に提供できない、あるいは利用者が当該サイトのすべてのページにアクセスできないかも知れないことなど。

「E コマース（電子商取引）」を目的とするサイトは、商取引のトランザクションに入ることがあるとすれば、そのタイミングと、その時には利用者の特別な承諾を得ることを、利用者に明確に知らせておく責務がある。

プライバシー

⑤利用者のプライバシー保護に配慮する。

医療関連目的でインターネットを利用する人たちは、自らが提供した個人情報の機密が保持されることを求める権利を有する。なかでも、個人の医療情報は極めて慎重に扱う必要があり、不適切な形で開示された場合、重大な結果を招く恐れがある。利用者を保護するために、個人情報の収集を行う各サイトは、以下の各点に留意するものとする。

・ 個人情報への不正アクセスや不正使用を防止するための妥当な措置を講じる。
  たとえば、利用者個人の医療や経済状況にかかる情報を含むすべてのトランザクションを対象とした、データの「暗号化」やパスワードによるファイル保護、適切なセキュリティ・ソフトウエアの使用など。

・ 提供した個人情報の確認や、必要に応じての更新・修正を、利用者が容易に行えるようにする。

・ 個人情報の用途を追跡するための適切な機構を採用する。
  たとえば、データを誰がいつ閲覧したかを示す「追跡監査」を行うなどによって。

・ サイトが利用者の個人情報の保管する方法や保管の期限を告知する。
・ 個人情報の「抹消」（利用者の身元が明らかにするための氏名や E メールアドレス、その他のデータをファイルから削除）を行うと当該利用者への連絡ができなくなることを理解してもらう。

オンライン医療におけるプロフェッショナリズム

⑥患者および顧客に対して基本的な倫理的責務を果たす。また、患者および顧客がオンライン医療の限界を理解

オンラインで、個人向けの特定の医療ケアや助言を提供する医師や看護婦、薬剤師、療法士、その他すべての医療従事者は、以下の各項を遵守するものとする。

するよう、啓発する。

- 患者や顧客と直接関わる関係において、医療従事職に適用される倫理綱領を遵守する。
- いかなる危害も与えないこと。
- 患者および顧客の利益を最優先する。
- 患者の機密を保護する。
- 医療従事者の役割や提供されるサービスに対する患者及び顧客の理解に影響を及ぼすと思われるスポンサーシップや金銭的なインセンティブ、その他の情報を明確に開示する。
- オンラインでの医療相談が有償の場合はその対価、また支払い方法を明確に開示する。
- 医療従事者の免許および処方に適用される、準拠法を含む当該管轄領域の関連法規を遵守する。

インターネットは患者の医療ニーズを満たすための強力な支援ツールになりうるが、利用者はその限界についても理解しておく必要がある。インターネット上で医療サービスを提供する医療従事者は、以下の各点を明瞭かつ正確に掲示するものとする。

- 患者や顧客に自分の身元を明らかにし、開業場所および認定資格を公開する。
- オンラインでの特定のやり取りの諸条件を提示する。
  たとえば、医療従事者が特定の疾患について一般的な助言を行うのか、もしくは、患者や顧客に対する特定の忠告や専門医への紹介を行うのかどうか。あるいは医療従事者が特定の条件下で薬剤を処方できるか否か、また、処方する意志があるか否か。
- 患者や顧客の特定の状況を理解し、患者や顧客が当該地域で入手可能な医療資源（人的・物的）の在り処を知る支援をすべく、誠意をもって努める。

たとえば、患者や顧客が自分の居住地域で特定の治療が受けられるか、居住地域外の医療機関でしか受けることができないかにより、どこに行くかを決める手助けをする。

・ 必要に応じ、フォローアップ・ケアについて明確な指導をする。

オンラインで個人向けの医療サービスや助言を提供する医療従事者は、以下の点に留意するものとする。

・ オンラインでの診断および治療アドバイスの制約について、明瞭正確に説明しておく。

たとえば、オンラインの医療従事者は患者を直接診察することができないため、患者は自分が必要とする医療のニーズをできる限り明確に述べることが重要である点を、医療提供者は強調する必要がある。

・ オンラインでの診察が医療提供者との対面のやり取りに代用できる場合と、代用できない場合、また、代用してはならない場合について、オンライン医療を受ける患者(e-patients)の理解を促す。

責任あるパートナー

⑦関連する組織およびサイトが信頼に足ることを確実にする

インターネット上で活動する組織および個人は、信頼できる個人または組織とのみパートナー関係を結ぶことができる点について、インターネット利用者は確信がもてなければならない。営利、非営利にかかわらず、サイトは以下の各点を確実にするものとする。

・ スポンサーやパートナー、その他の関連団体がそれぞれの準拠法を遵守し、当該サイトと同一の倫理規範を支持していくよう、適宜努力をする。

・ 利用者がキーワードやトピックに関する特定の情報について検索した時、その結果が表示される方法に対し、現在のスポンサーあるいは将来的なスポンサーが影響を及ぼすものでない点を強調する。

また、サイトは利用者に以下の点を明示するものとする。

・ 他のサイトへのリンクが参考程度のものか、あるいは、他のサイトの推奨を伴うものか。

・ 利用者が当該サイトを去って他のサイトに移動する時点を明確に示す。
たとえば、 移行を示す画面の使用など。

アカウンタビリティ（誠意ある対応）

⑧利用者がサイトに確実にフィードバックを行える機会を提供する。

インターネット上で医療関連の情報や製品、サービスを提供する組織および個人が、利用者の関心を真摯に受け止め、それぞれのサイトがその行動面において倫理的に問題がないことをより確実にすべく誠意をもって努力していることを、利用者が確信できるようにする必要がある。eHealth サイトは、以下の点を遵守するものとする。

• サイトまたはサービスの運営者、ならびに、サイトまたはサービスの管理責任者に連絡を取る方法を利用者に明示する。
たとえば、問題に対応する権限を有する特定の管理者または顧客サービス担当者に連絡を取る方法。

• 利用者がサイトやその提供する情報、製品、サービスの質に関するフィードバックを行う際に使いやすいツールを提供する。

・ 利用者からの苦情を速やかに把握し、迅速かつ適切に対応する。

また、eHealth サイトは、リンク設定がされている外部サイトを含め、サイトの商業的または非商業的なパートナーや関連団体が、法律や倫理規範に違反している可能性があると思われた場合、サイト管理者や顧客サービス担当者にその旨を通知してもらえるよう利用者に奨励するものとする。

そして、
サイトは eHealth 倫理コードの遵守をモニターしなければならない。

eHealth サイトは自己モニタリングの方針を利用者に明確に説明し、サイトの運営スタッフおよび関連団体による創意ある問題解決の取り組みを奨励するものとする。

eHealth Ethics Initiatives 2000　禁無断転載
